# Isolation, characterization and selection of indigenous *Bradyrhizobium* strains with outstanding symbiotic performance to increase soybean yields in Mozambique

**DOI:** 10.1016/j.agee.2017.06.017

**Published:** 2017-08-01

**Authors:** Amaral Machaculeha Chibeba, Stephen Kyei-Boahen, Maria de Fátima Guimarães, Marco Antonio Nogueira, Mariangela Hungria

**Affiliations:** a Universidade Estadual de Londrina (UEL), Dept. Agronomy, C.P. 10.011, 86.057-970, Londrina, PR, Brazil; b Embrapa Soja, C.P. 231, 86001-970, Londrina, PR, Brazil; c International Institute of Tropical Agriculture (IITA), P.O. Box 709, Nampula, Mozambique

**Keywords:** Biological nitrogen fixation, Symbiosis, Promiscuous soybean, *Rhizobium*, 16S rRNA, BOX-PCR, MLSA

## Abstract

Soybean inoculation with effective rhizobial strains makes unnecessary the use of N-fertilizers in the tropics. A frequently reported problem is the failure of the inoculant strains to overcome the competition imposed by indigenous rhizobial populations. The screening of indigenous rhizobia, already adapted to local conditions, searching for highly effective strains for use as inoculants represents a promising strategy in overcoming inoculation failure. The objective of this study was to isolate and characterize indigenous rhizobia and to identify strains that hold potential to be included in inoculant formulations for soybean production, with both promiscuous and non-promiscuous soybean cultivars, in Mozambican agro-climatic conditions. A total of 105 isolates obtained from nodules of promiscuous soybean grown at 15 sites were screened for N_2_-fixation effectiveness in the greenhouse along with five commercial strains. Eighty-seven isolates confirmed the ability to form effective nodules on soybean and were used for genetic characterization by rep-PCR (BOX) and sequencing of the 16S rRNA gene, and also for symbiotic effectiveness. BOX-PCR fingerprinting revealed remarkable genetic diversity, with 41 clusters formed, considering a similarity level of 65%. The 16S rRNA analysis assigned the isolates to the genera *Bradyrhizobium* (75%) and *Agrobacterium/Rhizobium* (25%). Great variability in symbiotic effectiveness was detected among the indigenous rhizobia from Mozambique, with ten isolates performing better than the commercial strain *B. diazoefficiens* USDA 110, the best reference strain, and 51 isolates with lower performance than all reference strains. Thirteen of the best isolates from the first greenhouse trial were evaluated, along with the five commercial strains, in two promiscuous (TGx 1963-3F and TGx 1835-10E) and one non-promiscuous (BRS 284) soybean cultivars in a second greenhouse trial. In general the promiscous soybeans responded better to inoculation. The 13 isolates were also characterized for tolerance to acidity and alkalinity (pH 3.5 and 9.0, respectively), salinity (0.1, 0.3 and 0.5 mol L^−1^ of NaCl) and high temperatures (35, 40 and 45 °C) *in vitro*. Five isolates, three (Moz 4, Moz 19 and Moz 22) belonging to the superclade *B. elkanii* and two (Moz 27 and Moz 61) assigned to the superclade *B. japonicum,* consistently showed high symbiotic effectiveness, suggesting that the inoculation with indigenous rhizobia adapted to local conditions represents a possible strategy for increasing soybean yields in Mozambique. Phylogenetic position of the five elite isolates was confirmed by the MLSA with four protein-coding housekeeping genes, *dnaK*, *glnII*, *gyrB* and *recA.*

## Highlights

• Biological nitrogen fixation (BNF) is a key process for soybean production in Africa.
• The selection of elite African indigenous soybean *Bradyrhizobium* strains is a feasible strategy.
• Eighty-seven isolates were obtained from soybean nodules in Mozambique.
• Isolates fit into the *Bradyrhizobium* (75%) and *Agrobacterium-Rhizobium* (25%) clades.
• Five *Bradyrhizobium* isolates with outstanding symbiotic performance were obtained.

## 1 Introduction

Soybean [*Glycine max* (Linnaeus) Merrill] stands out as the best-bet legume to feed the growing world population, projected to be between 9.6 and 12.3 billion in 2100, with much of the increase expected to happen in Africa (Gerland et al., 2014, UN, 2015). With approximately 40% seed protein and 20% seed oil content (Arslanoglu et al., 2011), soybean is an excellent source of food, fodder and biofuels. Like most legumes, soybean has the ability to reduce atmospheric nitrogen (N_2_) to a biologically usable ammonia (NH_3_), in association with bacteria collectively known as rhizobia (Singleton et al., 1992, Giller, 2001), obviating the need for N fertilizers. This is particularly important in Africa, where the predominantly subsistence farmers can hardly afford the limited available agricultural inputs (Singleton et al., 1992, Maingi et al., 2006, Chianu et al., 2011). In Mozambique, the demand for soybean has increased notably in recent years (Lava Kumar et al., 2011, Cunguara et al., 2012), to supply the growing poultry industry and for exportation (Dias and Amane, 2011, Muananamuale et al., 2012).

Many reports have established that when soybean is grown for the first time outside Southeast Asia, its centre of origin and domestication (Giller, 2001, Li et al., 2010), it yields poorly (Pulver et al., 1985, Maingi et al., 2006, Abaidoo et al., 2007, Chianu et al., 2011, Giller et al., 2011, Grönemeyer et al., 2014), presumably due to the lack of co-evolved rhizobial strains in soils abroad (Mpepereki et al., 2000, Giller, 2001, Parr, 2014). Successful introduction of soybean into new regions is, therefore, dependent on inoculation with exotic rhizobia.

In Africa, where economic and farmer scale problems have limited the possibility of distribution of commercial inoculants for decades, a practical alternative to the dependence on inoculation for developing countries was proposed. Researchers at the International Institute of Tropical Agriculture (IITA) developed soybean TGx genotypes (Tropical *Glycine* cross), known as promiscuous cultivars, due to their capacity of forming effective symbiotic relationships with a broad range of rhizobia indigenous to African soils (Pulver et al., 1985, Sanginga et al., 1996, Abaidoo et al., 2007, Tefera, 2011). Considerable evidence, nevertheless, indicates that, in many locations, indigenous rhizobial populations are either not effective, or do not occur in sufficient number to meet N demand of promiscuous cultivars (Sanginga et al., 2000, Okogun and Sanginga, 2003, Abaidoo et al., 2007, Klogo et al., 2015). This suggests that it is safer to inoculate soybean with effective rhizobial strains than relying on resident strains of unknown potential (Giller, 2001, Osunde et al., 2003).

Soybean response to inoculation is influenced by a number of soil factors including temperature (Al-Falih, 2002, Niste et al., 2013), N availability (Thies et al., 1991, Singleton et al., 1992), salinity (Tu, 1981, Zahran, 1999, Niste et al., 2013), pH (Giller, 2001, Al-Falih, 2002), and indigenous rhizobial population (Thies et al., 1992, Osunde et al., 2003). Very often, inoculant strains must compete with populations of indigenous or naturalized rhizobia, frequently not effective, but highly competitive and already adapted to the environment (Streeter, 1994; Al-Falih, 2002; Grönemeyer et al., 2014). It is broadly believed that inoculation responses are more likely to occur when there are less than 10 cells of indigenous or naturalized rhizobia per gram of soil (Thies et al., 1991, Thies et al., 1992, Sanginga et al., 1996, Sessitsch et al., 2002, Okogun and Sanginga, 2003), or when a substantial component of the population is not effective (Brockwell et al., 1995, Osunde et al., 2003). However, in Brazil, soybean responses to reinoculation in soils with over 10^3^ cells and even 10^6^ cells g^−1^ of soil have been reported (Hungria et al., 1998, Hungria et al., 2006a, Hungria et al., 2013, Hungria and Mendes, 2015).

The occurrence of indigenous strains compatible with promiscuous soybean cultivars and with high symbiotic effectiveness in Africa (Abaidoo et al., 2000, Abaidoo et al., 2007, Sanginga et al., 2000, Musiyiwa et al., 2005, Klogo et al., 2015, Gyogluu et al., 2016) suggests that effective, competitive and locally adapted strains can be selected for use in inoculants for soybean. Recently published evidence from Mozambique indicates that indigenous rhizobia capable of establishing effective symbiosis with both promiscuous and non-promiscuous soybean cultivars do occur in the country (Gyogluu et al., 2016). The objective of this study was to isolate and characterize indigenous rhizobia, and to identify strains that hold potential to be included in inoculant formulations for soybean production under Mozambican agro-climatic conditions, for both promiscuous and non-promiscuous soybean cultivars.

## 2 Material and methods

### 2.1 Site and soil description, and nodule sampling

To trap indigenous rhizobia, seven promiscuous soybean cultivars were sown at 15 sites within research stations owned by IITA in Manica (4), Nampula (2), Tete (6) and Zambézia (3) provinces (Table 1 and Supplementary Fig. S1), which represent the main soybean production region in Mozambique. Selected fields had no known history of soybean cultivation or rhizobia inoculation. The climate types, based on the Köppen-Geiger climate classification system, were *Cwa* (dry winter, wet summer) in Manica, Tete and Zambézia, and *Aw* (savanna) in Nampula. Soil types, according to the FAO/UNESCO soil classification, were Rhodic Ferralsols in Manica and Zambézia, Ferric Luvisols in Nampula, and Orthic Ferralsols in Tete.

**Table 1:** Location, soil characteristics and soybean cultivars from where indigenous rhizobia were obtained in Mozambique.

Sampling sites			Cultivars	Number of isolates sampled^a^	Soil characteristics								
	Coordinates				Silt	Sand	Clay	SOM^b^	pH^c^	P^d^	K	Ca	Mg
	South	East			g kg^−1^			g dm^−3^ CaCl_2_		mg dm^−3^			
Tete province													
Ntengo	14°32.8′	34°11.2′	TGx 1485-ID	7	133	537	330	21.9	5.3	2.2	219	1240	234
Ntengo	14°35.8′	34°11.2′	TGx 1835-10E	5	173	420	407	22.8	6.3	8.0	786	1740	428
Ntengo	14°32.8′	34°11.2′	TGx 1937-1F	5	114	459	427	24.1	6.1	15.6	259	2250	255
Nkhame	14°37.5′	33°58.9′	TGx 1904-6F	7	134	719	147	25.2	5.5	19.1	122	734	132
Nkhame	14°37.6′	33°58.9′	TGx 1740-2F	7	133	640	227	34.7	7.2	29.9	269	4080	223
Nkhame	14°37.6′	33°58.9′	TGx 1908-8F	7	114	699	187	16.6	5.4	16.0	84	604	159

Zambézia province													
Ruace	15°14.1′	36°41.3′	TGx 1740-2F	5	113	817	70	18.1	5.3	28.5	148	722	116
Ruace	15°14.0′	36°41.4′	TGx 1987-38F	5	114	797	89	14.7	5.2	31.0	126	584	97
Mutequelesse	15°19.2′	36°42.7′	TGx 1908-8F	7	114	799	87	17.9	5.5	25.5	150	735	116

Nampula province													
Muriaze	15°16.4′	39°18.8′	TGx 1937-1F	6	56	664	280	27.4	5.9	3.9	219	1450	173
Muriaze	15°16.4′	39°19.0′	TGx 1908-8F	7	74	699	227	38.6	7.3	5.4	205	3800	148

Manica province													
Sussundenga	19°18.9′	33°14.5′	TGx 1485-ID	5	36	897	67	16.2	5.5	16.5	63	490	74
Sussundenga	19°18.9′	33°14.5′	TGx 1740-2F	6	73	797	130	22.9	5.5	18.5	285	836	167
Zembe	19°15.8′	33°23.1′	TGx 1904-6F	6	136	577	287	37.8	5.3	11.6	305	728	164
Zembe	19°15.9′	33°23.0′	TGx 1485-ID	2	114	799	87	27.2	5.5	7.9	176	779	158

a Only those (in total of 87) used for genetic and symbiotic characterization are considered.

b SOM, Soil Organic Matter = 1.724 × soil organic carbon.

c pH CaCl_2_ was estimated based on the equation pH (CaCl_2_) = pH (H_2_O) × 0.923 − 0.373 (Ahern et al., 1995).

d Organic P.

Sixty days before sowing, 20 soil subsamples were collected at each site from the 0–20 cm layer for chemical and granulometry analyses (Table 1). For chemical analysis, samples were oven dried at 60 °C for 48 h and sieved (2 mm). Soil pH was determined in H_2_O (1/2; soil/H_2_O), 1 h after shaking (Peech, 1965). Calcium, Mg, K, Al and P were determined after extraction with Mehlich-3 (0.2 mol L^−1^ C_2_H_4_O_2_, 0.25 mol L^−1^ N_2_H_4_O_3_, 0.015 mol L^−1^ NH_4_F, 0.013 mol L^−1^ NHO_3_, and 0.001 mol L^−1^ C_10_H_16_N_2_O_8_) (1/10; soil/solution) (Sims, 1989) using inductively coupled plasma optical emission spectroscopy (ICP-OES). Soil organic carbon (SOC) was determined based on the Walkley-Black chromic acid wet oxidation method (Walkley and Black, 1934) and soil organic matter (SOM) was determined considering, SOM = 1.724 × SOC. Soil particle sizes were determined by the hydrometer method (Kilmer and Alexander, 1949). Nodules were sampled in March–April 2013. At each site, five to ten nodules per plant were harvested from five randomly selected healthy plants, about 50 days after sowing. A minimum of 15 nodules were randomly selected from each sampling site.

### 2.2 Bacteria isolation from root nodules

At Embrapa Soja (Brazil) laboratory, undamaged nodules were immersed in 70% (v/v) C_2_H_2_O for 10 s, and then in 10% (v/v) NaClO for 4 min. They were subsequently rinsed six times with sterile H_2_O to remove traces of NaClO. The isolation and purification of bacteria were performed as previously reported (Vincent, 1970). The surface-sterilized nodules were crushed individually and the nodule suspension was streaked onto plates containing yeast-mannitol agar (YMA) medium (Vincent, 1970) modified to contain 5 g L^−1^ of mannitol and 0.00125% Congo red (w/v). After confirming the purity of each single type of colony, the isolates were maintained on YMA slants at 4 °C for short-term storage. For long-term storage isolates were maintained on YM with 30% (w/v) glycerol at both –80 °C and –150 °C, and lyophilized. A total of 256 isolates were obtained and of these, seven were randomly selected from each of the 15 sampling sites, resulting in 105 isolates used in this study.

### 2.3 Genetic characterization

#### 2.3.1 DNA extraction

Isolates and reference strains were grown at 28 °C on a rotary shaker operating in the dark at 90 cycles per minute for three to seven days and DNA was extracted with DNeasy Blood & Tissue kit (QIAGEN^®^, Germany). Mini-gels (8 cm × 10 cm) of 1.0% (w/v) agarose and 0.5 × Tris-acetate/EDTA (TAE) were employed in electrophoresis at 60 V for 35 min, using DNA Mass^®^ Ladder, to confirm DNA purity. Gels were then stained with C_21_H_20_BrN_3_, visualized and photographed under UV light.

#### 2.3.2 PCR amplification with primer BOX A1R

The DNA of 87 selected isolates and of commercial/reference strains was amplified with BOX A1R (5′-CTACGGCAAGGCGACGCTGACG-3′, Invitrogen^®^ Life Technologies^®^, São Paulo, Brazil) (Versalovic et al., 1994). The final volume of the PCR reaction was a 25 μL mixture containing sterile milli-Q H_2_O, 13.8 μL; dNTPs (1.5 mmol L^−1^ of each), 5.0 μL; MgCl_2_ 50 mmol L^−1^, 1.5 μL; buffer 10× (500 mmol L^−1^ KCl; 100 mmol L^−1^ Tris–HCl, pH 8.3), 2.5 μL; BOX A1R primer (50 pmol μL^−1^), 1.0 μL; Taq DNA polymerase (5 U μL^−1^), 0.2 μL; sample DNA 50 ng μL^−1^, 1.0 μL.

The reaction was carried out in a thermocycler (Eppendorf^®^ Mastercycler Gradient^®^, Hamburg, Germany), as follows: one cycle of denaturation at 95 °C for 7 min; 30 cycles of denaturation at 94 °C for 1 min, annealing at 53 °C for 1 min, and extension at 65 °C for 8 min; one cycle of final extension at 65 °C for 16 min; and a final soak at 4 °C. PCR fragments were separated by horizontal electrophoresis on a 1.5% agarose gel (20 cm × 25 cm), at 120 V, for 6 h. A 1 kb DNA marker (Invitrogen^®^) was placed at both ends and in the middle of each gel.

After electrophoresis, gels were stained with C_21_H_20_BrN_3_, visualized and photographed under UV light, with a digital camera (Kodak^®^, China). The obtained BOX A1R-PCR products were grouped considering a level of similarity of 65% in the cluster analysis with UPGMA (Unweighted Pair Group Method with Arithmetic Mean) algorithm and Pearsońs correlation. All analyses were performed with the software Bionumerics^®^ 7.5 (Applied Mathematics, Sint-Martens-Latem, Belgium).

#### 2.3.3 Amplification of the DNA region coding for the 16S rRNA and protein-coding dnaK, glnII, gyrB and recA genes

The DNAs of 41 isolates selected from the BOX-PCR analysis were amplified for 16S rRNA, as indicated in Supplementary Table S1. In addition, the DNAs of five promising isolates were amplified for the *dnaK, glnII, gyrB* and *recA* genes, as indicated in Supplementary Table S1**.** The obtained amplified products were purified using a PureLink^®^ Quick PCR Purification Kit (Invitrogen^®^ by Life Technologies^®^, Löhne, Germany). The concentration of the samples was verified in 1% (w/v) agarose gels and stored at –20 °C until further processing.

#### 2.3.4 *Sequencing of the 16S rRNA,* dnaK*,* glnII*,* gyrB *and* recA *genes*

Primers and amplication conditions for sequencing the 16S rRNA, *dnaK, glnII, gyrB* and *recA* genes are shown in Supplementary Table S1. PCR products were purified with PureLink^®^ Quick PCR Purification Kit and sequenced with a 3500XL Genetic Analyzer (Hitachi^®^, Applied Biosystems^®^, California, USA). The obtained gene sequences were deposited in the GenBank and the accession numbers are indicated in the figures and in Supplementary Tables S2 and S3.

#### 2.3.5 Sequence analysis

All phylogenetic analyses were performed with the software MEGA^®^ 6 (Tamura et al., 2013). Pairwise and multiple sequence alignments were generated with CLUSTAL W (Larkin et al., 2007). The best model of sequence evolution was established with Modeltest (Posada and Crandall, 1998) based on the lowest Bayesian Information Criterion (BIC) score (Schwarz, 1978). Phylogenetic trees were reconstructed by the maximum-likelihood (ML) and neighbor-joining (NJ) statistical methods and the robustness of branching was estimated with 1000 bootstrap replicates (Felsenstein, 1985). The degree of similarity between nucleotide sequences was determined with the software Bioedit^®^ 7.2.5 (Hall, 1999). Since ML and NJ methods generated very similar topologies only ML based phylograms are presented.

16S rRNA gene – based phylogenetic trees were reconstructed with sequences of representative isolates from Mozambique, reference strains used in inoculants and type strains retrieved from the GeneBank. Protein-coding *dnaK, glnII, gyrB* and *recA* gene – based phylogenetic trees were also reconstructed with the five isolates from Mozambique that had the best performance in the greenhouse trials, along with the reference and *Bradyrhizobium* type strains employed in the 16S rRNA gene analysis. The accession numbers of the employed bacteria are indicated in parenthesis in the phylogenetic trees and are summarized in Supplementary Tables S2 and S3.

### 2.4 Characterization of symbiotic properties

#### 2.4.1 Isolates, reference strains and soybean cultivars

The 105 indigenous rhizobial isolates from Mozambique were screened for symbiotic N_2_-fixation effectiveness in a greenhouse along with four reference strains used in commercial inoculants in Brazil, *B. japonicum* SEMIA 5079 (=CPAC 15), *B. diazoefficiens* SEMIA 5080 (=CPAC 7), *B. elkanii* strains SEMIA 587 and SEMIA 5019 (=29w), and a strain broadly used in commercial inoculants in Africa, *B. diazoefficiens* USDA 110. The trial was performed with soybean cultivar BRS 133 (Brazilian, non-transgenic, genealogy FT-Abyara X BR 83–147), a typical modern genotype of non-promiscuous nodulation.

Subsequently, a second greenhouse trial was performed, with 13 of the most effective isolates identified in the first trial, in addition to the five reference strains. In this trial, two promiscuous (African, TGx 1963-3F and TGx 1835-10E) and one non-promiscuous (Brazilian, BRS 284, non-transgenic, genealogy Mycosoy–45 × Suprema) soybean cultivars were employed. Two non-inoculated control treatments were included in both trials, control with (control + N, 80 mg of KNO_3_ plant^−1^ week^−1^) and without (control – N).

#### 2.4.2 Inocula preparation, trial management and experimental design

Each bacterium was grown in YM medium for five days and then adjusted to a concentration of 10^9^ cells mL^−1^. Soybean seeds were surface-sterilized as described in Section 2.2. Sowing was carried out in December 2013 and June 2015, for the first and second trials, respectively.

Four seeds were sown in each of the pre-sterilized Leonard jars (Vincent, 1970) containing a mixture of sand and pulverized coal (1/2, v/v) and N-free autoclaved nutrient solution with pH adjusted to 6.6–6.8 (Andrade and Hamakawa, 1994). Each seed was individually treated with 1 mL inoculum, equivalent to 1.2 10^6^ cells seed^−1^. Jars were thinned to contain two seedlings at five and ten days after emergence (DAE), for the first and second trials, respectively. All through the trials, plants were kept with an adequate volume of N-free solution. Air temperature and relative humidity inside the greenhouse were daily recorded at 09h00 and 15h00 throughout the trials. In the first trial, the daily mean air temperatures at 09h00 and 15h00 were 26.0 ± 1.9 and 30.3 ± 2.9 °C (mean ± SD), respectively, whereas the daily mean air relative humidity records were 67.0 ± 9.6 and 54.6 ± 7.1%, respectively. In the second trial, the daily mean air temperatures at 09h00 and 15h00 were 22.1 ± 1.6 and 25.0 ± 2.8 °C, respectively, whereas the daily mean air relative humidity records were 69.1 ± 6.3 and 66.1 ± 8.1%, respectively.

The first trial was laid out in a randomized complete block design (RCBD) with four replicates. For the second trial, a factorial 20 × 3 (18 inoculants + non-inoculated control without N + non-inoculated control with N × three soybean cultivars) fitted in RCBD with four replications was used.

#### 2.4.3 Evaluation of nodulation, plant growth and nitrogen accumulation in shoots

The plants were harvested at 35 and 41 DAE, respectively, for the first and second trials, both at R2 [reproductive stage, one open flower at one of the two uppermost nodes on the main stem with a completely developed leaf]. Stems were cut at the cotyledonary node separating plant shoots from roots. Shoots were placed in labeled paper bags, with each bag containing shoots from only one jar, and dried at 50 °C for 72 h. Samples were then weighed to determine shoot dry weight (SDW) and ground (18 mesh) to quantify total N accumulation in shoots (TNS) by the salicylate green method (Searle, 1984). Roots and adhering rooting medium were dislodged and washed over 1 mm mesh screen. Soil particles adhering to the roots were carefully rinsed off with a gentle stream of H_2_O. Roots and nodules were placed in paper bags and dried for 72 h at 50 °C and weighed to determine root dry weight (RDW) (only in the second trial). Nodules were then detached from the roots, counted, to determine nodule number (NN), and allowed to dry further before weighing to determine nodule dry weight (NDW). At a later stage, relative effectiveness (RE) was determined as the percentage of SDW of plants supplied with N (Control + N).

#### 2.4.4 Statistical analyses

As most data failed to meet ANOVA assumptions, nonparametric statistics were performed to analyze data from the first trial. Spearmen’s rank correlation was used to assess relationships among soybean nodulation, plant growth and production variables with software Statistica^®^ 10.0 (StatSoft, 2011). Relationships among isolates and sampling sites were explored with principal component analysis using software Analyse-it^®^ (Analyse-it Software Ltd, Leeds, UK). In the second trial, original TNS data were transformed with x^½^ prior to ANOVA testing to attain Gaussian data distribution and homoscedasticity. When differences among treatments were detected (ANOVA, p < 0.05), Tukey’s test (p < 0.05) was performed to compare treatment means. The software Sisvar^®^ (Ferreira, 2011) was used for data analyses.

### 2.5 Morphophysiological characterization

The ability of the best 13 isolates to grow under stressed conditions *in vitro*, including salinity, acidity, alkalinity and high temperature, was assessed as described elsewhere (Chen et al., 2002). The isolates and reference strains were grown in the dark in tubes with 100 μL of YM medium with pH adjusted to 6.8–7.0, at 28 °C on a rotary shaker operating at 90 cycles per minute and optical density (OD) was measured on the seventh day in a spectrophotometer (Spectronic Genesys^®^2, Spectronic Instruments^®^, New York, USA) at λ  = 600 nm as control readings. To assess tolerance to salinity, the samples were grown in YM medium supplemented with 0.1, 0.3 and 0.5 mol L^−1^ of NaCl. Sensitivity to acidity or alkalinity was evaluated in YM medium adjusted to pH 3.5 or 9.0. The ability to grow under high temperatures was assessed at 35, 40 and 45 °C. All evaluations were made with three replicates and tolerance was expressed as the percentage of OD in relation to the control treatment.

## 3 Results

### 3.1 Isolates used in the study

Of the 105 isolates obtained from soybean nodules collected in Mozambique, 18 did not nodulate the non-promiscuous soybean cultivar BRS 133 and, as the objective of the study was to select isolates able to nodulate both promiscuous and non-promiscuous cultivars, they were not considered in the analyses. Hence, the screening for N_2_-fixation and the genetic characterization were performed with 87 isolates (Table 2).

**Table 2:** Nodule number (NN, n° plant^−1^) and dry weight (NDW, mg plant^−1^), shoot dry weight (SDW, g plant^−1^), total N accumulation in shoots (TNS, mg plant^−1^) and relative effectiveness (RE, %) of soybean, cultivar BRS 133, inoculated with 87 isolates from Mozambique, for each BOX-PCR cluster and five reference strains, *B. elkanii* SEMIA 587 and SEMIA 5019, *B. japonicum* SEMIA 5079, and *B. diazoefficiens* SEMIA 5080 and USDA 110. Trial performed under greenhouse conditions in Londrina, Brazil, and plants harvested at 35 days after emergence.

Cluster a	Isolates	Source b	Species name c	NN d	NDW	SDW	TNS	RE e
1	29, 31	5	*Bradyrhizobium* sp.	79.9	456.66	3.1	59.47	73.0
2	27^f^	4	*Bradyrhizobium* sp.	65.5	493.08	5.2	146.15	129.5
3	97	14	*Bradyrhizobium* sp.	68.8	352.67	3.6	92.65	88.0
4	95^f^	14	*Bradyrhizobium* sp.	68.4	397.79	4.2	102.18	98.9
5	10	2	*Rhizobium* sp.	7.0	29.14	0.8	6.51	18.2
6	38^f^	6	*Rhizobium* sp.	73.0	541.83	4.0	100.71	96.4
7	85, 87, 88	13	*Rhizobium* sp.	29.1	121.02	1.7	38.00	40.0
8	52, 53	8	*Bradyrhizobium* sp.	22.2	79.05	1.0	12.60	22.9
9	56	8	*Bradyrhizobium* sp.	11.8	54.59	0.8	6.97	18.3
10	34	5	*Bradyrhizobium* sp.	29.1	236.00	2.1	30.74	48.8
11	81,82	12	*Bradyrhizobium* sp.	10.9	37.42	0.8	8.14	19.4
12	46, 48	7	*Bradyrhizobium* sp.	14.3	56.58	0.9	9.80	21.0
13	60, 61^f^	9	*Bradyrhizobium* sp.	77.3	419.79	4.7	130.59	112.0
14	37, 39^f^, 40^f^, 41	6	*Bradyrhizobium* sp.	75.5	555.01	4.7	124.22	109.4
	20, 22^f^, 23, 24^f^, 25	3,4						
15	26, 28, 30, 32, 33,	4,5	*Bradyrhizobium* sp.	75.7	519.38	5.2	132.27	120.5
	35, 57, 58, 62^f^, 63	5, 9						
16	15, 17^f^, 18, 19^f^	3	*Bradyrhizobium* sp.	96.0	546.24	5.2	121.29	122.9
17	1, 2, 3, 6^f^, 7	1	*Bradyrhizobium* sp.	76.4	438.13	4.3	91.59	100.9
18	4^f^, 5	1	*Bradyrhizobium* sp.	68.3	532.74	5.6	136.66	130.9
19	64, 65, 66, 67	10	*Bradyrhizobium* sp.	37.6	138.28	1.6	29.69	35.0
20	59	9	*Rhizobium* sp.	37.4	310.11	2.9	53.69	70.4
21	70	10	*Bradyrhizobium* sp.	38.0	161.78	1.5	32.01	34.9
22	86, 90, 91	13	*Rhizobium* sp.	17.1	86.63	1.1	16.56	25.9
23	55	8	*Rhizobium* sp.	11.4	55.44	0.9	10.98	21.2
24	73	11	*Rhizobium* sp.	23.6	63.90	0.9	12.94	21.6
25	78, 79, 80	12	*Bradyrhizobium* sp.	6.5	42.02	0.8	8.32	18.6
26	50	8	*Rhizobium* sp.	5.5	18.93	0.8	5.76	20.5
27	76, 77	11	*Bradyrhizobium* sp.	25.0	120.43	1.8	27.65	42.3
28	75	11	*Rhizobium* sp.	16.1	53.29	0.8	13.35	19.3
29	42	6	*Rhizobium* sp.	64.3	488.68	3.8	107.68	88.9
30	69, 92	10, 14	*Rhizobium* sp.	26.7	116.13	1.5	30.61	33.8
31	93	14	*Rhizobium* sp.	12.5	39.39	0.8	8.12	18.3
32	94, 100	14, 15	*Rhizobium* sp.	10.4	33.99	1.0	12.40	23.8
33	8	2	*Agrobacterium* sp.	38.3	241.22	2.7	40.80	64.0
34	9, 11	2	*Bradyrhizobium* sp.	21.2	105.63	1.5	20.96	34.9
35	14	2	*Bradyrhizobium* sp.	10.0	27.53	1.1	9.20	24.3
36	44	7	*Bradyrhizobium* sp.	9.8	19.71	0.7	4.74	17.2
37	71, 72, 74	11	*Bradyrhizobium* sp.	21.2	83.10	1.4	17.88	32.7
38	43, 45	7	*Bradyrhizobium* sp.	19.4	48.37	0.9	9.18	20.8
39	36	6	*Rhizobium* sp.	9.4	29.68	0.8	7.19	19.1
40	96	14	*Bradyrhizobium* sp.	10.3	41.55	0.9	13.87	22.2
41	99	15	*Rhizobium* sp.	31.4	70.24	1.0	13.14	23.3

Reference strains								
USDA 110		USA	*B. diazoefficiens*	61.5	408.81	5.4	140.20	127.9
SEMIA 587		Brazil	*B. elkanii*	59.8	265.06	3.9	84.60	93.9
SEMIA 5019		Brazil	*B. elkanii*	56.4	513.58	5.0	119.28	118.8
SEMIA 5079		Brazil	*B. japonicum*	73.4	350.91	3.4	89.16	81.5
SEMIA 5080		Brazil	*B. diazoefficiens*	81.6	391.29	3.7	77.31	86.4

a Phylogenetic cluster as defined by BOX-PCR analysis (Fig. 1).

b Sampling sites: 1–Ntengo_1_; 2–Ntengo_2_; 3–Ntengo_3_; 4–Khame_1_; 5–Khame_2_; 6–Khame_3_; 7–Ruace_1_; 8–Ruace_2;_ 9–Mutequelesse; 10–Muriaze_1_; 11–Muriaze_2_; 12–Sussundenga_1_; 13–Sussundenga_2_; 14–Zembe_1_; 15–Zembe_2_.

c Based on the 16S rRNA gene analysis (Fig. 2, Fig. 3).

d Values of each isolate represent the average of four replications.

e Expressed as the percentage of shoot dry weight of plants supplied with N (Control + N).

f Isolates selected for the second greenhouse trial.

### 3.2 Genetic characterization

#### 3.2.1 BOX A1R-PCR genomic fingerprinting

DNA profiles with an average of 200 bands and sizes between 200 and 5000 bp were obtained for the 87 isolates and five soybean bradyrhizobial reference strains, after performing PCR with the primer BOX A1R. The isolates were grouped in 41 phylogenetic clusters (Fig. 1). Thirty-two of the 41 clusters (78%) were composed of less than three isolates, three clusters (14, 16 and 19) were composed of four isolates and there was a cluster (15) with 15 isolates (Fig. 1).

**Fig. 1:**
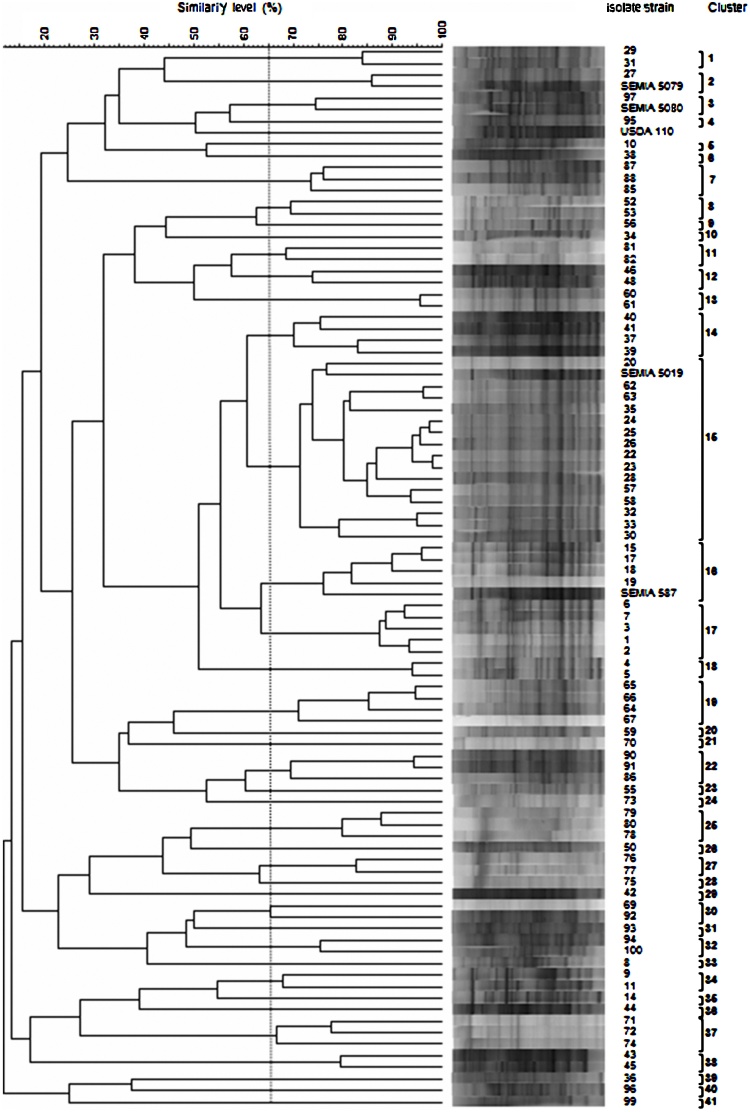
Dendrogram of 87 isolates from Mozambique and five reference strains used in commercial inoculants, *B. elkanii* SEMIA 587 and SEMIA 5019, *B. japonicum* SEMIA 5079, and *B. diazoefficiens* SEMIA 5080 and USDA 110 based on cluster analysis of BOX-PCR products using the UPGMA algorithm and Pearson correlation.

In general, isolates were unevenly distributed across sites. Ntengo_2_, Ruace_2_, Zembe_1_, Zembe_2_ were the most heterogeneous sites with 80% or more isolates clustered in different BOX-PCR groups (Fig. 1, Table 2). On the other hand, Ntengo_1_ (71%), Nkhame_1_ (71%) and Sussundenga_2_ (67%) were the most homogenous sampling sites with more than 65% of the isolates from the same cluster. The dendrogram also showed that the two *B. elkanii* reference strains clustered with 19 isolates from Mozambique, 15 in cluster 15 with strain SEMIA 5019 and four in cluster 16 with strain SEMIA 587. *B. japonicum* SEMIA 5079 and *B. diazoefficiens* SEMIA 5080 were each joined to one isolate, while *B. diazoefficiens* strain USDA 110 was not clustered to any isolate (Fig. 1).

#### 3.2.2 Phylogeny based on the 16S rRNA gene

**Fig. 2:**
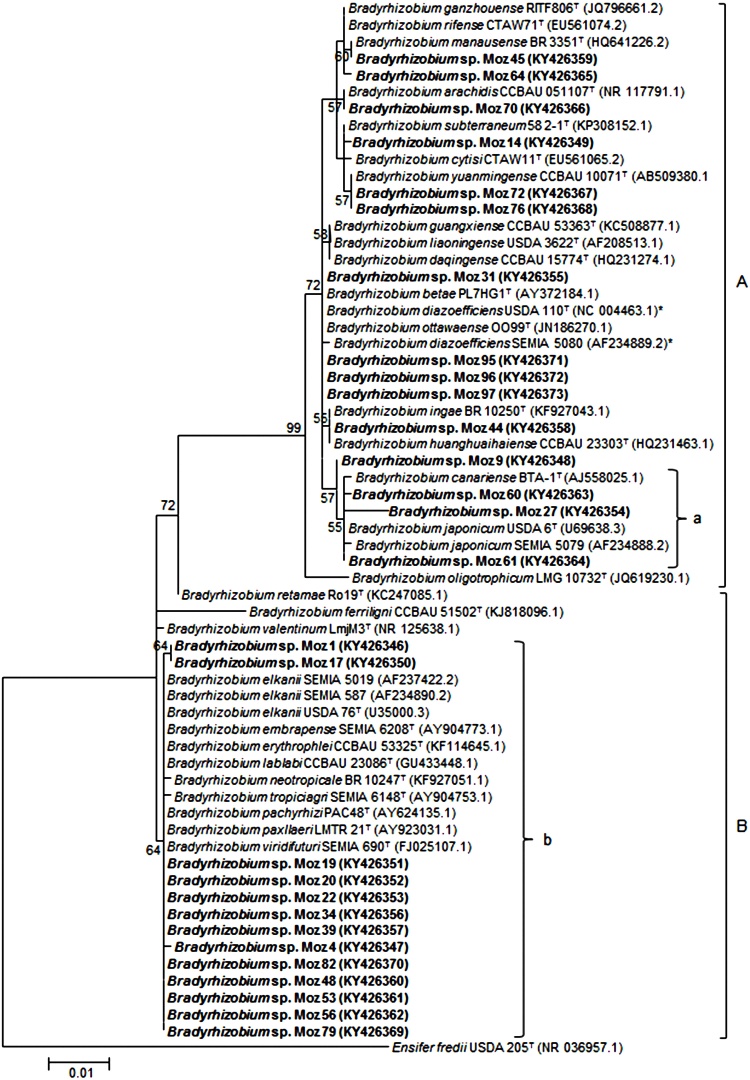
Maximum – likelihood phylogeny based on 16S rRNA gene sequences (846 bp) showing the relationships among representative *Bradyrhizobium* isolates from Mozambique (in bold) with *Bradyrhizobium* type (^T^) and reference strains used in commercial inoculants, *B. elkanii* SEMIA 587 and SEMIA 5019, *B. japonicum* SEMIA 5079, and *B. diazoefficiens* SEMIA 5080 and USDA 110 (with an asterix). *Ensifer fredii* USDA 205^T^ was inluded as an outgroup. The evolutionary history was inferred using the nearest-neighbor-interchange method. Only bootstrap confidence levels >55% are indicated at the internodes. The scale bar indicates 1 substitution per 100 nucleotides. A and B represent clades *B. japonicum* and *B. elkanii*, respectively; a and b represent the clustering of the five best nitrogen fixer strains from Mozambique.

**Fig. 3:**
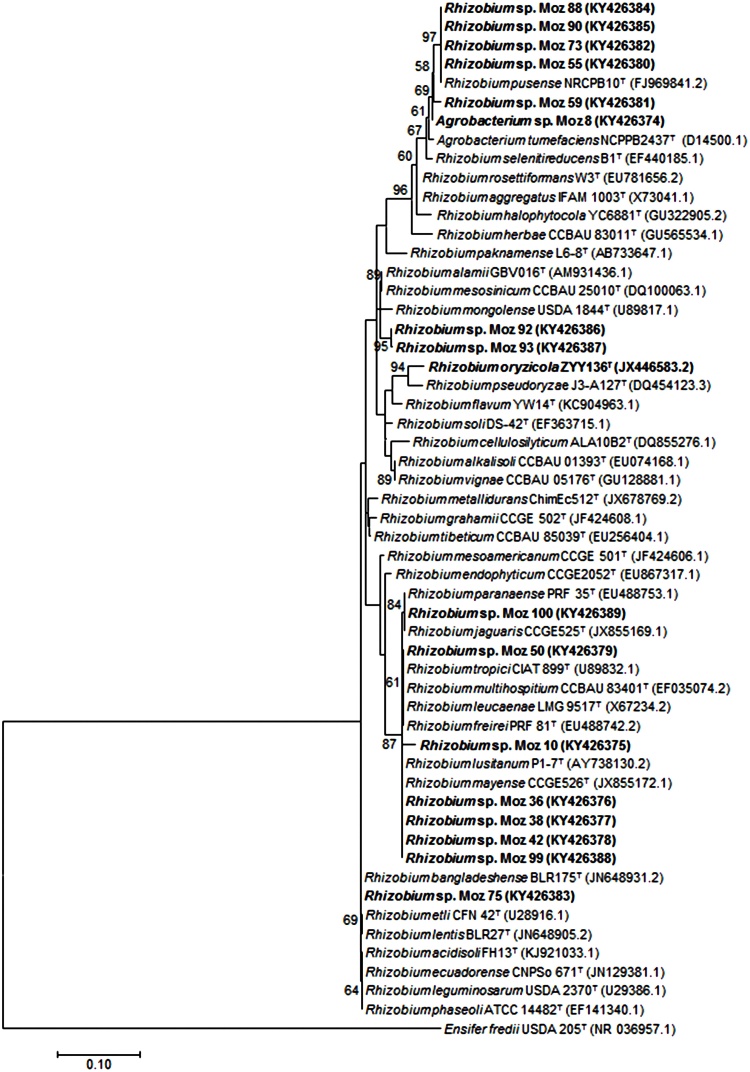
Maximum – likelihood phylogeny based on 16S rRNA gene sequences (621 bp) showing the relationships among representative *Agrobacterium* – *Rhizobium* isolates from Mozambique (in bold) with *Agrobacterium* – *Rhizobium* type strains (^T^). *Ensifer fredii* USDA 205^T^ was inluded as an outgroup. Only bootstrap confidence levels > 55% are indicated at the internodes. The scale bar indicates 1 substitution per 10 nucleotides.

In the analysis of the 16S rRNA gene, the majority of the isolates were assigned to the *Bradyrhizobium* (75% of the isolates), and the remaining to the *Agrobacterium/Rhizobium* (25%) genera (Table 2)*.* In many cases, the BOX A1R-PCR (Fig. 1) analysis was consistent with the 16S rRNA gene phylogeny (Figs. 2 and 3). Several isolates, such as Moz 1, Moz 4, Moz 17, Moz 20 and Moz 39, which were clustered close to the reference strains *B. elkanii* SEMIA 587 and SEMIA 5019 in the BOX-PCR analysis (Fig. 1), were positioned in the superclade of *B. elkanii* in the 16S rRNA phylogram (Fig. 2). In both BOX-PCR and 16S rRNA analyses, isolates Moz 95 and Moz 97 were clustered with reference strains *B. diazoefficiens* SEMIA 5080 and USDA 110^T^ (Figs. 1 and 2).

In the 16S rRNA *Bradyrhizobium* phylogram, two superclades were observed, superclade A, of *B. japonicum,* and superclade B, of *B. elkanii* (Fig. 2). Isolates from Mozambique were distributed in the two superclades (Fig. 2), sharing high nucleotide identity with the majority of type/reference strains (Supplementary Table S4). The high nucleotide identity of the 16S rRNA gene recorded even for strains belonging to different species of *Bradyhrizobium* indicated that other genes were required to provide depth of the analysis at the species level. A biogeographic pattern was observed, as *Bradyrhizobium* was present in all sites, except for Sussundenga_2_ and Zembe_2_, and this was the only genus recorded at six (Nteng_1_, Ntengo_3_, Nkhame_1_, Nkhame_2_, Ruace_1_, Sussundenga_1_) out of the 15 sampled sites (Table 2).

In the genus *Agrobacterium/Rhizobium*, isolates Moz 55, Moz 73 and Moz 90 clustered closely in both BOX-PCR (Fig. 1) and 16S rRNA analyses (Fig. 3), and were grouped with strain *Rhizobium pusense* NRCPB10^T^ with a bootstrap value of 97% (Fig. 3). The genus *Agrobacterium/Rhizobium* was represented in 50% of the sampled isolates at Zembe_1_ and was exclusively recorded at Sussundenga_2_ and Zembe_2_ (Table 2).

#### 3.2.3 Multilocus sequencing analysis (MLSA) of dnaK, glnII, gyrB and recA genes

For the five isolates with outstanding symbiotic performance (from Section 3.3), a better phylogenetic definition was achieved by the MLSA analysis with four housekeeping genes, *dnaK, glnII, gyrB* and *recA*. Each single-gene based phylogeny showed the five strains from Mozambique separated in two groups (Supplementary Figs. S2 to S5). In the first group, strains Moz 27 and Moz 61 shared 100%, 100%, 99.2% and 99.2% of nucleotide identity in the *dnaK, glnII, gyrB* and *recA* phylogenies, respectively (Supplementary Table S4), and were clustered tightly (96–99% bootstrap support) with *B. japonicum* USDA 6^T^. In the second group, Moz 4, Moz 19 and Moz 22 shared 99.5–100%, 99.3–99.7%, 95.7–100% and 99.7–100% similarities in the *dnaK, glnII, gyrB* and *recA* phylogenies, respectively, with *B. elkanii* USDA 76^T^ (Supplementary Table S4), also sharing high bootstrap support (91–99%) (Supplementary Figs. S2 to S5), except in the *gyrB* phylogeny, where strain Moz 4 was separated from this group (Supplementary Fig. S4).

**Fig. 4:**
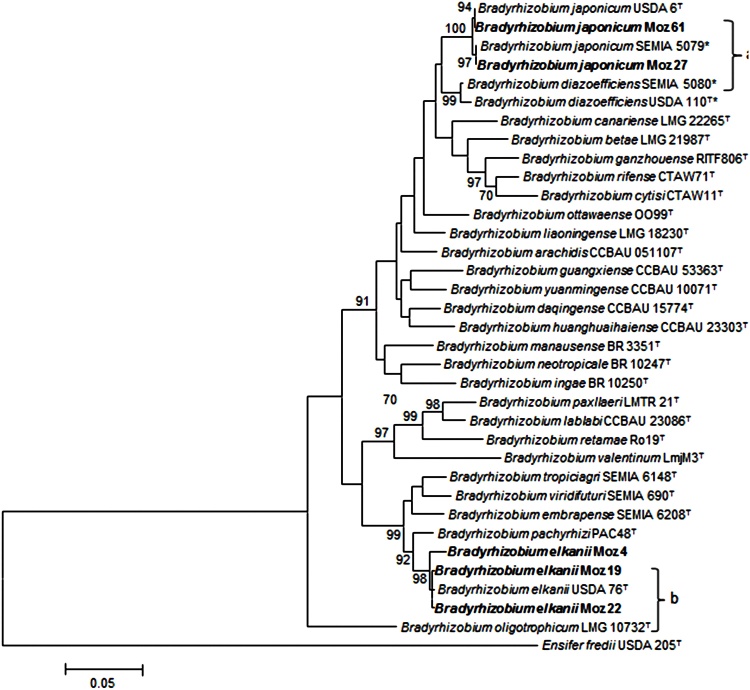
Maximum – likelihood phylogeny based on concatenated gene sequences [*dnaK* (223 bp), *glnII* (480 bp), *gyrB* (419 bp) and *recA* (375 bp)] showing the relationships among five rhizobial isolates from Mozambique (in bold) with type (^T^) and reference strains used in commercial inoculants, *B. elkanii* SEMIA 587 and SEMIA 5019, *B. japonicum* SEMIA 5079, and *B. diazoefficiens* SEMIA 5080 and USDA 110 (with an asterix). *Ensifer fredii* USDA 205^T^ was inluded as an outgroup. Only bootstrap confidence levels >70% are shown at the internodes. The scale bar indicates 5 substitutions per 100 nucleotides; a and b represent the clustering of the five best nitrogen fixer strains from Mozambique.

In the analysis of the concatenated genes, strains Moz 27 and Moz 61 shared 99.5% of nucleotide identity (Supplementary Table S4) and in the phylogenetic tree formed a well supported cluster (100% of bootstrap) with *B. japonicum* USDA 6^T^ (Fig. 4). Likewise, strains Moz 4, Moz 19 and Moz 22 shared 98.5–99.7% of nucleotide identity of the concatenated genes (Supplementary Table S4) and clustered with high bootstrap support (98%) with *B. elkanii* USDA 76^T^ (Fig. 4).

### 3.3 Symbiotic performance

#### 3.3.1 First trial

Nonparametric Spearman Rank analyses revealed positive and highly significant correlations between NN and NDW (r = 0.91, *p <* 0.001), NDW and SDW (r = 0.88, *p <* 0.001), NDW and TNS (r = 0.89, *p <* 0.001), SDW and TNS (r = 0.94, *p <* 0.001), and between SDW and RE (r = 0.93). Considering that SDW has been suggested as the best variable for assessing symbiosis (Haydock and Norris, 1980, Hungria and Bohrer, 2000, Souza et al., 2008), the high correlation between SDW and RE, and the practicality of using RE, this variable was used to make the general symbiotic assessment of the 87 isolates from Mozambique.

Great variability in symbiotic effectiveness was detected among the indigenous rhizobia from Mozambique with ten isolates performing better than USDA 110, the best reference strain, and 51 isolates with inferior symbiotic effectiveness than all the reference strains (Supplementary Table S5). Thirty-seven isolates had outstanding symbiotic performance, as indicated by RE > 80%, a similar performance to that of the reference strains (Table 2; Supplementary Tables S5 and S6). There was an uneven geographical distribution of very effective isolates across sampling sites, with high proportions found in only six out of the 15 sampling sites, Ntengo_1_ (100% = all isolates had RE > 80%), Ntengo_3_ (100%), Nkhame_1_ (100%), Nkhame_2_ (57%), Nkhame_3_ (86%) and Mutequelesse (86%) (Supplementary Tables S5 and S6).

A total of 40 isolates had low symbiotic performance, as shown by RE < 40% (Supplementary Tables S5 and S6). High proportions of these isolates were recorded in nine sampling sites, Ntengo_2_ (60%), Ruace_1_ (100%), Ruace_2_ (100%), Muriaze_1_ (83%), Muriaze_2_ (86%), Sussundenga_1_ (100%), Sussundenga_2_ (83%) and Zembe_2_ (100%) (Supplementary Tables S5 and S6).

**Fig. 5:**
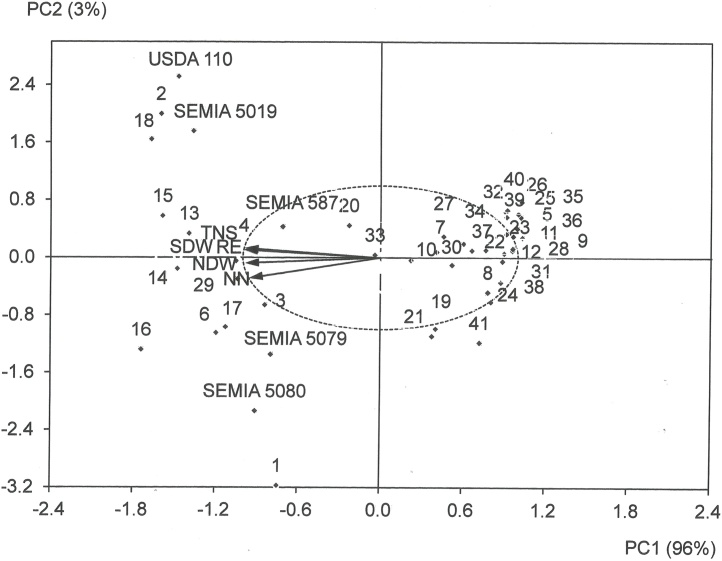
Principal component analysis exploring the symbiotic performance of the representative isolates from Mozambique and five reference strains, *B. elkanii* SEMIA 587 and SEMIA 5019, *B. japonicum* SEMIA 5079, and *B. diazoefficiens* SEMIA 5080 and USDA 110 based on nodule number (NN) and dry weight (NDW), shoot dry weight (SDW), total N accumulated in shoots (TNS) and relative effectiveness (RE). Numbers represent phylogenetic clusters as defined by BOX-PCR analyses (Fig. 1).

The symbiotic effectiveness of the 41 phylogenetic clusters, based on the BOX-PCR results, is presented in Table 2. Eleven clusters (2, 3, 4, 6, 13, 14, 15, 16, 17, 18 and 29) had RE > 80% and 24 clusters had RE < 40%. Principal component analyses were performed to explore the symbiotic performance of the representative isolates (Fig. 5) and their biogeographic distribution across the sampling sites (Supplementary Fig. S6). The analyses considered two components that together explained 99% of the variation in NN, NDW, SDW, TNS and RE. The 11 phylogenetic clusters with RE > 80% are located on the left side along with the reference strains, whereas the 24 poorly effective clusters are positioned on the right side of the graph (Fig. 5). Similarly, the sampling sites from where large proportions of isolates with RE > 80% were recorded are shown on the left side and sites with high proportion of isolates with RE < 40% are in the inferior and superior quadrants on the right side (Supplementary Fig. S6).

Interestingly, isolates of the same phylogenetic cluster tended to show similar symbiotic performance. For example, the RE variation among the highly effective clusters was as follows, cluster 13: mean = 112.0%, SD = 6.7% (n = 2, range = 107.3–116.8%); cluster 14: mean = 109.4%, SD = 12.4% (n = 4, range = 92.3–119.8%); cluster 15: mean = 120.5%, SD = 14.9% (n = 15, range = 84.6–138.4%) (Supplementary Table S5). The variations in RE among the worst three poorly effective phylogenetic clusters were as follows, cluster 12: mean = 21.0%, SD = 8.0% (n = 2, range = 15.3–26.7%); cluster 25: mean = 18.6%, SD = 2.9% (n = 3, range 15.6–21.5%); and cluster 38: mean = 20.8%, SD = 3.2% (n = 2, range = 18.5–23.0%).

#### 3.3.2 Second trial

The thirteen best performing isolates in the first trial were selected for a second greenhouse trial with two promiscuous (TGx 1963-3F and TGx 1835-10E) and one non-promiscuous (BRS 284) soybean cultivars. Significant differences were observed among the soybean cultivars in terms of root dry weight (RDW), with TGx 1963-3F recording significantly (p < 0.05) higher mean than TGx 1835-10E and BRS 284 (Table 3). Among the reference strains, USDA 110 was the most effective, resulting in the highest RDW. Considering the effect across cultivars, isolates Moz 38, Moz 40 and Moz 95 had the lowest means. TGx 1963-3F was revealed to be more promiscuous than TGx 1835-10E as it had significantly higher SDW (p < 0.05) with more isolates (Moz 17, Moz 22 and Moz 95 and SEMIA 587). TGx 1835-10E responded to inoculation with isolates Moz 38, Moz 61 and Moz 95 with significantly (p < 0.05) lower SDW than BRS 284. The symbiotic performance of isolates Moz 38 and Moz 61 on the promiscuous cultivars resulted in significantly (p < 0.05) lower SDW than the non-promiscuous BRS 284. Promiscuous cultivars responded to inoculation with isolates Moz 19 and Moz 40 with improved (p < 0.05) growth, compared to the non-promiscuous cultivar. In general, TGx 1963-3F had better (p < 0.05) growth than TGx 1835-10E and BRS 284 (Table 3).

**Table 3:** Root dry weight (RDW, g plant^−1^) and shoot dry weight (SDW, g plant^−1^) of two promiscuous (TGx 1963-3F and TGx 1835-10E) and one non-promiscuous (BRS 284) soybean cultivars inoculated with 13 indigenous rhizobial isolates from Mozambique and five reference strains, *B. elkanii* SEMIA 587 and SEMIA 5019, *B. japonicum* SEMIA 5079, and *B. diazoefficiens* SEMIA 5080 and USDA 110. Trial conducted under greenhouse conditions in Londrina, Brazil, and plants harvested at 41 days after emergence.

Isolate/ Strain	RDW				SDW			
	TGx 1963	TGx 1835	BRS 284	Mean	TGx 1963	TGx 1835	BRS 284	Mean
Moz 4	0.91 ^Aab^^a^	0.74 ^Ba−d^	0.73 ^Bab^	0.79 ^abc^	2.19 ^Aa^^a^	1.91 ^Aa−d^	1.96 ^Aa−d^	2.02 ^abc^
Moz 6	0.86 ^Aab^	0.68 ^Ba−e^	0.65 ^Bab^	0.73 ^b−f^	1.95 ^Aa^	1.65 ^ABb−e^	1.44 ^Bde^	1.68 ^de^
Moz 17	0.76 ^Abcd^	0.60 ^Bdef^	0.72 ^ABab^	0.69 ^c−f^	2.07 ^Aa^	1.49 ^Bde^	1.80 ^ABbcd^	1.79 ^b−e^
Moz 19	0.81 ^Aa−d^	0.78 ^Aa−d^	0.72 ^Aab^	0.77 ^a−e^	2.14 ^Aa^	2.09 ^Aabc^	1.67 ^Bcde^	1.97 ^a−d^
Moz 22	0.99 ^Aa^	0.79 ^Ba−d^	0.76 ^Bab^	0.85 ^a^	2.26 ^Aa^	1.80 ^Ba−d^	1.83 ^Ba−d^	1.96 ^a−d^
Moz 24	0.81 ^Aa−d^	0.70 ^Aa−d^	0.81 ^Aa^	0.77 ^a−e^	1.93 ^Aa^	1.60 ^Ab−e^	1.77 ^Abcd^	1.77 ^b−e^
Moz 27	0.85 ^Aab^	0.81 ^Aabc^	0.78 ^Aab^	0.81 ^ab^	2.08 ^Aa^	2.13 ^Aab^	1.97 ^Aa−d^	2.06 ^ab^
Moz 38	0.47 ^Be^	0.46 ^Bf^	0.66 ^Aab^	0.53 ^h^	1.21 ^Bb^	1.11 ^Be^	1.62 ^Ade^	1.31 ^f^
Moz 39	0.84 ^Aabc^	0.79 ^Aa−d^	0.72 ^Aab^	0.79 ^abc^	2.03 ^Aa^	1.88 ^Aa−d^	1.75 ^Abcd^	1.88 ^b−e^
Moz 40	0.74 ^Abcd^	0.66 ^ABb−e^	0.60 ^Bb^	0.67 ^efg^	1.92 ^Aa^	1.72 ^Abcd^	1.17 ^Be^	1.60 ^ef^
Moz 61	0.79 ^Abcd^	0.66 ^Bb−e^	0.79 ^Aab^	0.75 ^a−e^	1.98 ^Ba^	1.85 ^Ba−d^	2.40 ^Aa^	2.08 ^ab^
Moz 62	0.78 ^Abcd^	0.84 ^Aab^	0.75 ^Aab^	0.79 ^abc^	1.80 ^Aa^	1.82 ^Aa−d^	1.78 ^Abcd^	1.80 ^b−e^
Moz 95	0.72 ^Abcd^	0.49 ^Bef^	0.69 ^Aab^	0.63 ^f−h^	1.91 ^Aa^	1.45 ^Bde^	1.92 ^Aa−d^	1.76 ^b−e^
USDA 110	0.74 ^Abcd^	0.87 ^Aa^	0.75 ^Aab^	0.79 ^abc^	2.21 ^Aa^	2.35 ^Aa^	2.25 ^Aab^	2.27 ^a^
SEMIA 587	0.63 ^Ade^	0.49 ^Bef^	0.61 ^ABb^	0.57 ^gh^	1.83 ^Aa^	1.43 ^Bde^	1.71 ^ABb−e^	1.66 ^de^
SEMIA 5019	0.71 ^Abcd^	0.64 ^Ac−f^	0.69 ^Aab^	0.68 ^d−g^	1.73 ^Aab^	1.55 ^Acde^	1.74 ^Abcd^	1.67 ^de^
SEMIA 5079	0.72 ^Abcd^	0.67 ^Ab−e^	0.71 ^Aab^	0.70 ^c−f^	1.84 ^Aa^	1.63 ^Ab−e^	1.64 ^Ade^	1.70 ^cde^
SEMIA 5080	0.66 ^Acde^	0.71 ^Aa−d^	0.79 ^Aab^	0.72 ^b−f^	1.75 ^Bab^	2.09 ^ABabc^	2.23 ^Aabc^	2.02 ^abc^
Mean	0.76 ^A^	0.69 ^B^	0.72 ^B^	0.72	1.93 ^A^	1.75 ^B^	1.81 ^B^	1.83
Control + N^b^	0.82	0.83	1.03		3.14	3.16	3.74	
Control − N^b^	0.35	0.30	0.37		0.60	0.58	0.66	
C.V. (%)				10.77				12.25

a Means of four replications and when followed by same letter, uppercase on the same line or lowercase on the same column, are not statistically different by Tukey’s test at p < 0.05.

b Not included in statistical analysis.

Inoculation resulted in significantly (p < 0.05) higher nodule number (NN) in the non-promiscuous cultivar BRS 284 than in the promiscuous TGx 1963-3F and TGx 1835-10E cultivars (Table 4). BRS 284 had greater (p < 0.05) NN when inoculated with isolates Moz 17, Moz 19, Moz 24, Moz 38 and Moz 62 than TGx 1963-3F and TGx 1835-10E. Inoculation with isolates Moz 38 and Moz 40 resulted in significantly higher (p < 0.05) NN than that of all the other isolates, except for Moz 22 and Moz 39. On average, TGx 1963-3F had significantly (p < 0.05) lower NDW than TGx 1835-10E and BRS 284 (Table 4). Both promiscuous cultivars had significantly (p < 0.05) higher NDW than BRS 284 when inoculated with isolate Moz 6, but had significantly (p < 0.05) lower NDW when inoculated with isolates Moz 24 and Moz 38. Isolates Moz 19, Moz 22, Moz 27, Moz 39 and Moz 62 had the highest NDW and outperformed (p < 0.05) all the reference strains (Table 4).

**Table 4:** Nodule number (NN, n° plant^−1^) and nodule dry weight (NDW, mg plant^−1^) of two promiscuous (TGx 1963-3F and TGx 1835-10E) and one non-promiscuous (BRS 284) soybean cultivars inoculated with 13 indigenous rhizobial isolates from Mozambique and five reference strains, *B. elkanii* SEMIA 587 and SEMIA 5019, *B. japonicum* SEMIA 5079, and *B. diazoefficiens* SEMIA 5080 and USDA 110 grown under greenhouse conditions in Londrina, Brazil, and harvested at 41 days after emergence.

Isolate/Strain	NN				NDW			
	TGx1963	TGx 1835	BRS 284	Mean	TGx 1963	TGx 1835	BRS 284	Mean
Moz 4	28.00 ^Abc^^a^	29.17 ^Aa−e^	35.75 ^Ac^	30.97 ^cd^	267.75 ^Aabc^^a^	259.27 ^Abc^	254.13 ^Aa−e^	260.38 ^bcd^
Moz 6	27.75 ^ABbc^	21.63 ^Bcde^	35.38 ^Ac^	28.25 ^cd^	275.42 ^Aab^	266.52 ^Abc^	176.05 ^Bg^	239.33 ^def^
Moz 17	28.38 ^Babc^	26.50 ^Bb−e^	40.50 ^Abc^	31.79 ^bcd^	254.62 ^Abcd^	234.68 ^Acde^	261.38 ^Aa−d^	250.23 ^cde^
Moz 19	23.75 ^Bc^	21.00 ^Bcde^	40.50 ^Abc^	28.42 ^cd^	253.33 ^Bbcd^	292.76 ^Aab^	254.83 ^Ba−e^	266.97 ^bc^
Moz 22	43.00 ^Aab^	44.75 ^Aa^	36.67 ^Ac^	41.47 ^ab^	307.52 ^Aa^	313.48 ^Aa^	286.46 ^Aab^	302.49 ^a^
Moz 24	31.13 ^Babc^	23.25 ^Bcde^	42.83 ^Abc^	32.40 ^bcd^	227.87 ^Bdef^	234.22 ^Bcde^	295.13 ^Aa^	252.41 ^cde^
Moz 27	29.38 ^Aabc^	32.63 ^Aa−d^	36.00 ^Ac^	32.67 ^bcd^	248.04 ^Bbcd^	299.65 ^Aab^	289.11 ^Aa^	278.93 ^ab^
Moz 38	35.75 ^Babc^	32.33 ^Ba−d^	62.83 ^Aa^	43.64 ^a^	140.04 ^Bh^	161.70 ^Bgh^	226.07 ^Ac−f^	175.94 ^hi^
Moz 39	28.13 ^Aabc^	38.00 ^Aabc^	39.00 ^Abc^	35.04 ^abc^	271.40 ^Bab^	299.22 ^Aab^	264.08 ^Babc^	278.23 ^b^
Moz 40	45.50 ^ABa^	34.22 ^Ba−d^	54.50 ^Aab^	44.74 ^a^	222.35 ^ABdef^	247.04 ^Acd^	202.10 ^Bfg^	223.83 ^f^
Moz 61	38.88 ^Aabc^	21.25 ^Bcde^	35.75 ^Ac^	31.96 ^bcd^	218.43 ^ABdef^	210.07 ^Bdef^	246.00 ^Ab−e^	224.83 ^f^
Moz 62	29.88 ^Babc^	27.17 ^Bb−e^	42.33 ^Abc^	33.13 ^bcd^	248.23 ^Bbcd^	308.83 ^Aa^	286.52 ^Aab^	281.19 ^ab^
Moz 95	31.25 ^ABabc^	21.25 ^Bcde^	37.00 ^Ac^	29.83 ^cd^	193.11 ^ABf^	175.12 ^Bf−h^	213.75 ^Aefg^	194.00 ^gh^
USDA 110	29.13 ^Aabc^	33.13 ^Aa−d^	31.75 ^Ac^	31.33 ^cd^	238.44 ^Ab−e^	247.07 ^Acd^	227.60 ^Ac−f^	237.70 ^def^
SEMIA 587	22.75 ^ABc^	14.00 ^Be^	34.25 ^Ac^	23.67 ^d^	151.51 ^ABgh^	138.38 ^Bh^	175.63 ^Ag^	155.17 ^i^
SEMIA 5019	30.38 ^ABabc^	24.25 ^Bcde^	37.63 ^Abc^	30.75 ^cd^	205.58 ^Bef^	201.78 ^Befg^	254.75 ^Aa−e^	220.70 ^f^
SEMIA 5079	24.50 ^Bc^	19.33 ^Bde^	38.13 ^Abc^	27.32 ^cd^	238.91 ^Ab−e^	232.74 ^Acde^	231.10 ^Ac−f^	234.25 ^ef^
SEMIA 5080	33.88 ^Aabc^	42.13 ^Aab^	35.75 ^Ac^	37.25^abc^	191.75 ^Bfg^	232.89 ^Acde^	222.30 ^Adef^	215.65 ^fg^
Mean	31.19 ^B^	28.11 ^C^	39.81 ^A^	33.03	230.79 ^B^	241.97 ^A^	242.61 ^A^	238.46
Control + N^b^	0.00	0.00	0.00		0.00	0.00	0.00	
Control − N^b^	0.00	0.00	0.00		0.00	0.00	0.00	
C.V. (%)				20.97				6.91

a Means of four replications and when followed by same letter, uppercase on the same line or lowercase on the same column, are not statistically different by Tukey’s test at p < 0.05.

b Not included in statistical analysis.

Soybean cultivars differed in N accumulated in shoots (TNS), with TGx 1963-3F fixing significantly (p < 0.05) more N than TGx 1835-10E and BRS 284 (Table 5). Inoculation with isolates Moz 19, Moz 40 and Moz 95 favored higher (p < 0.05) N accumulation in the promiscuous cultivars than in the non-promiscuous one, but inoculation with isolate Moz 38 and reference strains USDA 110 and SEMIA 5080 resulted in significantly lower TNS in the promiscuous cultivars. Isolates Moz 38, Moz 61 and Moz 95 were the best among the 13 tested. Overall, the promiscuous cultivars had significantly greater (p < 0.05) relative effectiveness (RE) than the non-promiscuous BRS 284 (Table 5). The inoculation with isolates Moz 19, Moz 27, Moz 39 and Moz 40 was more favorable (p < 0.05) to the promiscuous cultivars than to BRS 284. Considering all cultivars, isolates Moz 19, Moz 22, Moz 27 and Moz 61 had the best symbiotic performance among the indigenous rhizobia and were significantly superior (p < 0.05) than three of the four reference strains from Brazil. USDA 110 outperformed (p < 0.05) all the other reference strains, except SEMIA 5080, but was not statistically different to isolates Moz 19, Moz 22, Moz 27 and Moz 61. USDA 110 and isolates Moz 4, Moz 19, Moz 22, Moz 27 and Moz 61 had the highest SDW and RE in both the first (Supplementary Table S5) and second (Table 3, Table 5) trials. In contrast, isolate Moz 38 had the lowest RE among the 13 isolates tested in the two trials (Supplementary Table S5; Table 3, Table 5).

**Table 5:** Total nitrogen accumulation in shoots (TNS, mg plant^−1^) and relative effectiveness (RE, %) of two promiscuous (TGx 1963-3F and TGx 1835-10E) and one non-promiscuous (BRS 284) soybean cultivars inoculated with 13 indigenous rhizobial isolates from Mozambique and five reference strains, *B. elkanii* SEMIA 587 and SEMIA 5019, *B. japonicum* SEMIA 5079, and *B. diazoefficiens* SEMIA 5080 and USDA 110 grown under greenhouse conditions in Londrina, Brazil, in 2015, and harvested at 41 days after emergence.

Isolate/Strain	TNS				RE^c^			
	TGx 1963	TGx 1835	BRS 284	Mean^b^	TGx 1963	TGx 1835	BRS 284	Mean
Moz 4	54.69 ^Aab^^a^	44.52 ^ABb−f^	40.32 ^Bde^	46.51 ^d−g^	63.32 ^Aab^^a^	50.55 ^Bb−e^	46.23 ^Bbcd^	53.36 ^b−e^
Moz 6	52.16 ^Aabc^	33.55 ^Bef^	26.52 ^Bef^	37.41 ^h^	61.40 ^Aab^	45.38 ^Bc−g^	36.88 ^Bde^	47.89 ^def^
Moz 17	54.21 ^Aab^	40.74 ^Bc−f^	31.50 ^Bdef^	42.15 ^f−h^	65.50 ^Aab^	42.34 ^Bd−g^	38.23 ^Bde^	48.69 ^de^
Moz 19	53.47 ^Aabc^	54.35 ^Aa−d^	32.89 ^Bdef^	46.91 ^d−g^	68.10 ^Aab^	70.77 ^Aa^	41.62 ^Bcde^	60.16 ^ab^
Moz 22	56.21 ^Aab^	43.56 ^Bc−f^	36.21 ^Bde^	45.33 ^d−h^	73.26 ^Aa^	56.45 ^Ba−e^	46.61 ^Bbcd^	58.77 ^abc^
Moz 24	42.94 ^Abc^	38.66 ^Adef^	38.19 ^Ade^	39.93 ^f−h^	61.65 ^Aab^	45.73 ^Bc−g^	45.24 ^Bcde^	50.87 ^cde^
Moz 27	45.19 ^Aabc^	49.28 ^Aa−d^	43.46 ^Acd^	45.98 ^d−g^	66.11 ^Aab^	64.18 ^Aab^	49.76 ^Ba−d^	60.02 ^ab^
Moz 38	43.86 ^Babc^	33.44 ^Cef^	73.70 ^Aab^	50.33 ^c−f^	38.03 ^ABc^	32.52 ^Bg^	47.09 ^Abcd^	39.21 ^f^
Moz 39	53.91 ^Aab^	40.93 ^Bc−f^	33.81 ^Bdef^	42.88 ^e−h^	60.57 ^Aab^	57.80 ^Aa−d^	44.12 ^Bcde^	54.16 ^b−e^
Moz 40	53.40 ^Aabc^	39.85 ^Bdef^	23.18 ^Cf^	38.81 ^gh^	59.02 ^Aab^	47.37 ^Bc−g^	30.03 ^Ce^	45.47 ^ef^
Moz 61	60.14 ^Aa^	48.34 ^Ba−e^	67.28 ^Ab^	58.59 ^bc^	63.16 ^Aab^	52.41 ^Bb−e^	60.93 ^ABab^	58.83 ^abc^
Moz 62	45.50 ^Aabc^	38.38 ^Adef^	39.85 ^Ade^	41.24 ^f−h^	57.64 ^Ab^	52.38 ^ABb−e^	45.25 ^Bcde^	51.76 ^b−e^
Moz 95	57.23 ^Aab^	56.89 ^Aabc^	40.29 ^Bde^	51.47 ^cde^	63.89 ^Aab^	32.98 ^Cfg^	49.00 ^Ba−d^	48.62 ^de^
USDA 110	57.68 ^Bab^	61.94 ^Bab^	92.16 ^Aa^	70.59 ^a^	62.90 ^Aab^	70.32 ^Aa^	64.14 ^Aa^	65.79 ^a^
SEMIA 587	58.75 ^Aab^	40.20 ^Bdef^	59.44 ^Abc^	52.80 ^cd^	53.37 ^Abc^	41.13 ^Befg^	43.80 ^ABcde^	46.10 ^ef^
SEMIA 5019	37.42 ^ABc^	32.63 ^Bf^	45.12 ^Acd^	38.39 ^gh^	55.21 ^Ab^	48.57 ^ABb−f^	43.99 ^Bcde^	49.26 ^de^
SEMIA 5079	52.49 ^Aabc^	42.48 ^Ac−f^	44.02 ^Acd^	46.33 ^d−g^	59.18 ^Aab^	44.98 ^Bc−g^	41.23 ^Bcde^	48.46 ^de^
SEMIA 5080	45.90 ^Cabc^	66.38 ^Ba^	93.32 ^Aa^	68.53 ^ab^	55.76 ^Ab^	58.21 ^Aabc^	56.63 ^Aabc^	56.86 ^a−d^
Mean	51.40 ^A^	44.78 ^B^	47.85 ^B^	48.01	60.45 ^A^	50.78 ^B^	46.15 ^C^	52.46
Control + N^d^	93.83	91.51	122.75		100.00	100.00	100.00	
Control − N^d^	4.55	7.91	8.30		18.97	18.28	17.73	
C.V. (%)				6.31				11.86

a Means of four replications and when followed by same letter, uppercase on the same line or lowercase on the same column, are not statistically different by Tukey’s test at p < 0.05.

b Original data transformed with x½, to meet ANOVA assumptions.

c Expressed as the percentage of shoot dry weight of plants supplied with N (Control + N).

d Not included in statistical analysis.

Similarly to the observed in the first trial (Table 2), SDW (Table 3) was positively and significantly correlated with NDW (Table 4) (r = 0.38, p < 0.001), TNS (r = 0.73, p < 0.001) and RE (0.84, p < 0.001) (Table 5) in the second trial, but in general correlation coefficients were higher in the first trial.

#### 3.3.3 Tolerance to acidity/alkalinity, high temperature and salinity

The 13 best performing isolates in the first trial were also evaluated in relation to the ability to grow under stressed conditions and the results are summarized in Table 6. Most isolates grew well in YM supplemented with 0.1 mol L^−1^ of NaCl, with two isolates (Moz 38 and Moz 40) growing better than the control. However, only three (Moz 17, Moz 38 and Moz 40) and two (Moz 17 and Moz 40) isolates showed tolerance to 0.3 and 0.5 mol L^−1^ of NaCl, respectively. In relation to the tolerance to acidity/alkalinity, all isolates grew remarkably well in YM at pH 9.0, as shown by OD higher than 65% in relation to control, but ten isolates (77%) had growth inhibited at pH 3.5, as indicated by OD lower than 7%. While all isolates tolerated 35 °C, as shown by OD values greater than 40% in relation to control, ten isolates (77%) had inhibited growth at 40 °C, as indicated by OD below 9%, and only two isolates (Moz 17 and Moz 19) had OD higher than 10% at 45 °C. Isolates Moz 17 and Moz 38 were the most endurable as shown by OD values greater than 50% when grown in YM supplemented with 0.3 mol L^−1^ of NaCl and 40 °C. Among the reference strains, USDA 110 was the most sensitive and SEMIA 5019 was the most endurable (Table 6).

**Table 6:** Tolerance (in% of control OD readings) to salinity, acidity/alkalinity and high temperature of 13 indigenous rhizobial isolates from Mozambique and five reference strains, *B. elkanii* SEMIA 587 and SEMIA 5019, *B. japonicum* SEMIA 5079, and *B. diazoefficiens* SEMIA 5080 and USDA 110.

Isolate/Strain	Salinity (mol L^−1^ of NaCl)			Acidity/Alkalinity (pH)		High temperatures (°C)		
	0.1	0.3	0.5	3.5	9.0	35	40	45
Moz 4	56.9 a	2.2	0.0	0.5	79.2	61.3	5.1	2.5
Moz 6	97.7	7.5	9.6	31.0	108.3	82.1	8.8	5.7
Moz 17	81.5	51.2	16.5	10.5	70.6	68.5	65.7	10.4
Moz 19	41.7	5.7	0.0	6.5	65.1	60.3	20.0	10.3
Moz 22	27.4	2.9	0.0	1.2	101.6	75.0	7.3	2.2
Moz 24	27.5	2.6	0.0	0.6	104.2	81.3	4.2	2.2
Moz 27	40.6	4.3	0.0	1.6	87.8	51.6	6.3	2.5
Moz 38	124.3	97.5	0.0	14.8	118.5	68.5	55.4	0.6
Moz 39	76.0	5.4	0.0	1.9	96.4	76.0	6.0	2.5
Moz 40	172.9	71.1	47.9	0.0	73.7	72.9	4.8	1.0
Moz 61	12.8	3.3	0.0	0.0	103.4	41.5	5.5	0.2
Moz 62	52.5	2.1	0.0	0.1	75.8	60.8	3.4	1.8
Moz 95	4.5	3.2	0.0	0.2	95.0	49.5	6.6	0.0
USDA 110	2.4	2.8	1.3	1.6	86.3	37.5	4.8	0.0
SEMIA 587	6.0	3.3	3.7	0.8	90.4	71.6	6.3	0.1
SEMIA 5019	49.3	7.7	8.8	6.8	105.3	99.6	15.5	3.1
SEMIA 5079	70.7	5.1	5.2	4.0	95.4	75.4	5.2	3.3
SEMIA 5080	18.6	5.6	5.3	1.4	98.4	39.0	9.1	2.2

a Mean of three replications and values represent rhizobia sample growth measured as percentage of control optical density (OD) at 600 nm.

## 4 Discussion

A total of 87 indigenous isolates trapped by promiscuous soybean cultivars (TGx) from soils of Mozambique were studied. The isolates were assigned to the *Bradyrhizobium* (75%) and *Agrobacterium/Rhizobium* (25%) genera*.* Most (63%) of the *Bradyrhizobium* isolates clustered within the superclade *B. elkanii* and the remaining showed genetic relatedness to the superclade *B. japonicum*. *Bradyrhizobium* has been repeatedly reported among indigenous rhizobia in Africa. In a study conducted in Malawi, *B. elkanii* was the dominant species that formed nodules with soybean (Parr, 2014). A survey conducted in Kenya identified all indigenous rhizobia nodulating soybean as *B. elkanii* (Herrmann et al., 2014). In addition, a study conducted with indigenous rhizobia isolated from soybean in Benin, Cameroon, Ghana, Nigeria, Togo and Uganda revealed the genera *Bradyrhizobium* and *Rhizobium* as the most abundant, and *B. elkanii* and *B. japonicum* were the most common species identified (Abaidoo et al., 2000).

BOX-PCR (Fig. 1) and 16S rRNA (Fig. 2, Fig. 3) analyses were not always fully congruent. For example, isolates Moz 72 and Moz 76 clustered tightly together in the 16S rRNA but were far apart in the BOX-PCR analysis. Likewise, isolate Moz 96 clustered with reference strain *B. diazoefficiens* USDA 110 in the 16S rRNA phylogram, but exhibited weak genetic relatedness in the BOX-PCR analysis. Because in the BOX-PCR strains belonging to same species may be positioned in more than one cluster, the method is not a reliable source of primary evidence for inferring species or even genera (Hungria et al., 2006b, Menna et al., 2009). However, BOX-PCR is a robust method for detecting diversity among strains (Menna et al., 2009). It was noteworthy the high genetic diversity detected in our study, as indicated by the 41 BOX-PCR clusters formed, considering a 65% level of similarity. Furthermore, all isolates joined at final similarity level of less than 15%, confirming the great genetic diversity among the indigenous rhizobia trapped by soybean from soils of Mozambique.

While 16S rRNA is a precise tool for defining kingdom and genera, alone it is often inappropriate for inferring species (Stackebrandt and Goebel, 1994, van Berkum et al., 2002), especially in some genera, as is the case of *Bradyrhizobium*, where strains from different species showing more than 99% of similarity are often reported (Fox et al., 1992; Delamuta et al., 2013; Durán et al., 2014; Grönemeyer et al., 2014). In this study, high nucleotide identity (99.7–100%) was recorded between type strains of different species in the 16S rRNA analysis (Supplementary Table S4). Therefore, for the five elite isolates with the best symbiotic performance, a further genetic characterization was performed based on MLSA of protein-coding housekeeping genes. The MLSA analysis gave great support to the classification of Moz 27 and Moz 61 as *B. japonicum* and of Moz 4, Moz 19 and Moz 22 as *B. elkanii*, confirming the usefulness of this analysis in supporting phylogenetic and taxonomic studies.

The high variability in N_2_-fixation effectiveness and uneven distribution of symbiotically effective isolates among the sampling sites observed in our study corroborate evidence from elsewhere in Africa. Studies conducted in six (Abaidoo et al., 2000) and nine (Abaidoo et al., 2007) African countries have consistently reported both great variation in symbiotic effectiveness among indigenous rhizobial isolates sampled at the same sites and broad geographic distribution of effective isolates. This study contributes to evidence that indigenous rhizobia capable of establishing highly effective symbiosis with soybean do occur in Africa (Abaidoo et al., 2000, Abaidoo et al., 2007, Musiyiwa et al., 2005, Tefera, 2011, Youseif et al., 2014a, Klogo et al., 2015, Gyogluu et al., 2016) and confirms that the indigenous strains are also capable of nodulating non-promiscuous soybean cultivars (Klogo et al., 2015).

Soybean cultivars that nodulate freely with indigenous rhizobia, known as TGx, were developed to obviate the need for inoculation in Africa (Pulver et al., 1985, Tefera, 2011). In our study, the high proportion of very effective isolates recorded at Ntengo, Nkhame and Mutequelesse suggests that, at these sites, TGx cultivars may be successfully grown without inoculation, providing that the population sizes are large enough for an effective nodulation that supports the plant N demand.

*B. diazoefficiens* USDA 110 was the best and most consistent reference strain recording the highest SDW, TNS and RE in the first greenhouse trial, and outperforming the other strains in all variables in the second trial. This corroborates the evidence that strain USDA 110 has superior N_2_-fixation abilities (Singleton et al., 1985, Pazdernik et al., 1997, Youseif et al., 2014b, Hungria and Mendes, 2015, Agoyi et al., 2016). Moreover, in the second trial, USDA 110 recorded the highest performance in all cultivars, supporting previous evidence that this strain is effective with a large number of soybean cultivars (Pazdernik et al., 1997, Agoyi et al., 2016). These results also give high support to the decision of using this strain in soybean trials in the N2Africa project (http://www.n2africa.org/),together with elite local strains identified in each country.

Fast-growing rhizobia assigned as *Agrobacterium/Rhizobium* represented a large (25%) proportion of the studied isolates. *Agrobacterium* (Chen et al., 2000, Youseif et al., 2014b) and *Rhizobium* (Abaidoo et al., 2000, Hong et al., 2010, Alam et al., 2015) strains have been previously isolated from soybean nodules. Fast-growing rhizobia are believed to have a number of advantages including high competitiveness, facility for commercial production, easier establishment in the soil (Chatterjee et al., 1990) and high N_2_fixation capacity (Youseif et al., 2014b, Alam et al., 2015). In this study, however, fast-growing rhizobia were medium to poor symbionts (Supplementary Table S5). Moreover, isolate Moz 38, the best fast-growing rhizobia in the first trial (Supplementary Table S5), was the worst symbiont in the second trial (Table 5).

In general the promiscuous cultivars (TGx 1963-3F and TGx 1835-10E) responded markedly better to inoculation than the non-promiscuous one (BRS 284), as indicated by higher RDW and SDW (Table 3), TNS and RE (Table 5) with the majority of isolates, validating previous reports that TGx cultivars establish effective symbioses with a wide range of indigenous rhizobia (Musiyiwa et al., 2005, Pule-Meulenberg et al., 2011, Tefera, 2011, Gyogluu et al., 2016).

The different response to inoculation between the TGx cultivars observed in our study substantiates previous findings (Musiyiwa et al., 2005, Klogo et al., 2015, Agoyi et al., 2016, Gyogluu et al., 2016) and highlights the need for screening cultivars to determine the best inoculant–TGx combination. These trials may serve to decide whether inoculation is required. For example, from the four TGx cultivars tested in Mozambique, one of the three cultivars that did not respond to inoculation had 2.0 t ha^−1^ of grains exclusively attributed to symbiosis with indigenous rhizobia and was recommended for use by resource-poor farmers without inoculation (Gyogluu et al., 2016).

The results of rhizobia tolerance to stressed conditions reported here are in line with previous observations. In an evaluation of rhizobia isolated from soybean grown in Paraguay, SEMIAs 587, 5019 and 5080 were used as reference strains and also had OD values lower than 10% in relation to the control treatment when grown under high salinity (0.3 and 0.5 mol L^−1^ of NaCl), acid conditions (pH 3.5) or high temperatures (45 °C) (Chen et al., 2002). Twelve out of the 13 examined isolates were *Bradyrhizobium* and of these ten (83%) had inhibited growth under high temperatures, as indicated by OD values below 10%, at 40 °C (Table 6), corroborating with similar studies on slow-growing rhizobia (Chen et al., 2002, Youseif et al., 2014a). The observation that most (77%) of the 13 isolates had inhibited growth at pH 3.5, as indicated by OD values below 7% (Table 6), is consistent with the much higher pH values (5.2–7.3) of the sampling sites (Table 1) and supports previous observations that rhizobial optimum pH is neutral to moderately alkaline (Yadav and Vyas, 1971).

In conclusion, indigenous rhizobia isolated from nodules of soybean grown in Mozambique have been characterized. Large differences in the capacity to grow under stressing conditions including acidity/alkalinity, salinity and high temperature were observed. Isolates also exhibited high phylogenetic and symbiotic variability. Five elite isolates—*B. elkanii* Moz 4, Moz 19, Moz 22, and *B. japonicum* Moz 27 and Moz 61—consistently showed high N_2_-fixation effectiveness, suggesting that the inoculation with indigenous rhizobia already adapted to local conditions may represent an important strategy for increasing soybean yields in Mozambique. Multi-site field trials with those promising isolates will now be conducted to ascertain their superiority in fixing N_2_ in the presence of other indigenous and/or commercial strains.
