# Supplementary material for: Isolation, characterization and selection of indigenous Bradyrhizobium strains with outstanding symbiotic performance to increase soybean yields in Mozambique
Source: Agric Ecosyst Environ. 2017 Aug 1;246:291–305. doi: 10.1016/j.agee.2017.06.017 (PMC5521954; doi:10.1016/j.agee.2017.06.017)
Supplement: Supplementary file 1 [file mmc1.docx]

*Agriculture, Ecosystem & Environment*

**Isolation, characterization and selection of indigenous *Bradyrhizobium* strains with outstanding symbiotic performance to increase soybean yields in
Mozambique**

Amaral Machaculeha Chibeba, Stephen Kyei-Boahen, Maria de Fátima Guimarães, Marco Antonio Nogueira, Mariangela Hungria

**Supplementary Material**

**Table S1**

Primers and PCR conditions under which DNA was amplified.

| Primer | Sequence (5`- 3`) | Target gene (position) | PCR cycling | Reference |
| --- | --- | --- | --- | --- |
| TS*dnaK*2 | GTACATGGCCTCGCCGAGCTTCA | *dnaK* (1794–1772) | 1 min 94°C, 35 X (1 min 94 °C, 1 min | Stępkowski et al*.* (2003) |
| Ts*dnaK*3 | AAGGAGCAGCAGATCCGCATCCA | *dnaK* (1468–1490) | 62°C, 40 sec 72 °C) |  |
| TS*glnII*f | AAGCTCGAGTACATCTGGCTCGACGG | *glnII* (13–38) | 2 min 95°C, 35 X (45s 95°C, 30s 58°C, | Stepkowski et al*.* (2005) |
| TS*glnII*r | SGAGCCGTTCCAGTCGGTGTCG | *glnII* (681-660) | 1.5 min 72°C and 7 min 72°C. |  |
| *gyrB*343F | TTCGACCAGAAYTCCTAYAAGG | *gyrB* (343-364) | 5 min 95°C, 5X (2 min 94°C, 2 min |  |
| *gyrB*1043R | AGCTTGTCCTTSGTCTGCG | *gyrB* (1061-1043) | 58°C, 1 min 72°C) 28 X (30s 94°C, 1 min  58°C, 1 min 72°C and 5 min 72°C. | Martens et al*.* (2008) |
| TS*recA*f | CAACTGCMYTGCGTATCGTCGAAGG | *recA* (8-32) | 2 min 95°C, 35 X (45s 95°C, 30s 58°C | Stepkowski et al*.* (2005) |
| TS*recA*r | CGGATCTGGTTGATGAAGATCACCATG | *recA* (620-594) | 1.5 min 72°C and 7 min 72°C |  |
| fD1 | AGAGTTTGATCCTGGCTCAG | 16S rRNA (9-29) | 2 min 95°C, 30 X (15s 94°C, 45s 93°C, | Weisburg et al*.* (1991) |
| rD1 | CTTAAGGAGGTGATCCAGCC | 16S rRNA (1474-1494) | 45s 55°C, 2 min 72°C and 5 min 72°C. |  |

**Table S2**

Access numbers of the 16S rRNA sequences of type (^T^) and reference (*) strains, and isolates from Mozambique (in bold).

| Type/ Reference strain | Accession number | |
| --- | --- | --- |
| *Bradyrhizobium arachidis* CCBAU 051107^T^ | NR 117791.1 | |
| *B. betae* PL7HG1^T^ | AY372184.1 | |
| *B. canariense* BTA-1^T^ | AJ558025.1 | |
| *B. cytisi* CTAW11^T^ | EU561065.2 | |
| *B. daqingense* CCBAU 15774^T^ | HQ231274.1 | |
| *B. diazoefficiens* SEMIA 5080* | AF234889.2 | |
| *B. diazoefficiens* USDA 110^T^ * | NC 004463.1 | |
| *B. elkanii* SEMIA 5019* | AF237422.2 | |
| *B. elkanii* SEMIA 587* | AF234890.2 | |
| *B. elkanii* SEMIA 690^T^ | FJ025107.1 | |
| *B. elkanii* USDA 76^T^ | U35000.3 |  |
| *B. embrapense* SEMIA 6208^T^ | AY904773.1 | |
| *B. erythrophlei* CCBAU 53325^T^ | KF114645.1 | |
| *B. ferriligni* CCBAU 51502^T^ | KJ818096.1 | |
| *B. ganzhouense* RITF806^T^ | JQ796661.2 | |
| *B. guangxiense* CCBAU 53363^T^ | KC508877.1 | |
| *B. huanghuaihaiense* CCBAU 23303^T^ | HQ231463.1 | |
| *B. ingae* BR 10250^T^ | KF927043.1 | |
| *B. japonicum* SEMIA 5079* | AF234888.2 | |
| *B. japonicum* USDA 6^T^ | U69638.3 | |
| *B. lablabi* CCBAU 23086^T^ | GU433448.1 | |
| *B. liaoningense* USDA 3622^T^ | AF208513.1 | |
| *B. manausense* BR 3351^T^ | HQ641226.2 | |
| *B. neotropicale* BR 10247^T^ | KF927051.1 | |
| *B. oligotrophicum* LMG 10732^T^ | JQ619230.1 | |
| *B. ottawaense* OO99^T^ | JN186270.1 | |
| *B. pachyrhizi* PAC48^T^ | AY624135.1 | |
| *B. paxllaeri* LMTR 21^T^ | AY923031.1 | |
| *B. retamae* Ro19^T^ | KC247085.1 | |
| *B. rifense* CTAW71^T^ | EU561074.2 | |
| *B. subterraneum* 58 2-1^T^ | KP308152.1 | |
| *B. tropiciagri* SEMIA 6148^T^ | AY904753.1 | |
| *B. valentinum* LmjM3^T^ | NR 125638.1 | |
| *B. yuanmingense* CCBAU 10071^T^ | AB509380.1 | |
| *Ensifer fredii* USDA 205^T^ | NR 036957.1 | |
| *Agrobacterium tumefaciens* NCPPB2437^T^ | D14500.1 | |
| *R. acidisoli* FH13^T^ | KJ921033.1 | |
| *R. aggregatum* IFAM 1003^T^ | X73041.1 | |
| *R. alamii* GBV016^T^ | AM931436.1 | |
| *R. alkalisoli* CCBAU 01393^T^ | EU074168.1 | |
| *R. bangladeshense* BLR175^T^ | JN648931.2 | |
| *R. cellulosilyticum* ALA10B2^T^ | DQ855276. | |
| *R. ecuadorense* CNPSo 671^T^ | JN129381.1 | |
| *R. endophyticum* CCGE 2052^T^ | EU867317.1 | |
| *R. etli* CFN 42^T^ | U28916.1 | |
| *R. flavum* YW14^T^ | KC904963.1 | |
| *R. fredii* LMG 6217^T^ | X67231.1 | |
| *R. freirei* PRF 81^T^ | EU488742.2 | |
| *R. grahamii* CCGE 502^T^ | JF424608.1 | |
| *R. halophytocola* YC6881^T^ | GU322905.2 | |
| *R. herbae* CCBAU 83011^T^ | GU565534.1 | |
| *R. jaguaris* CCGE525^T^ | JX855169.1 | |
| *R. leguminosarum* USDA 2370^T^ | U29386.1 | |
| *R. lentis* BLR27^T^ | JN648905.2 | |
| *R. leucaenae* LMG 9517^T^ | X67234.2 | |
| *R. lusitanum* P1-7^T^ | AY738130.2 | |
| *R. mayense* CCGE526^T^ | JX855172.1 | |
| *R. mesoamericanum* CCGE 501^T^ | JF424606.1 | |
| *R. mesosinicum* CCBAU 25010^T^ | DQ100063.1 | |
| *R. metallidurans* ChimEc512 ^T^ | JX678769.2 | |
| *R. mongolense* USDA 1844^T^ | U89817.1 | |
| *R. multihospitium* CCBAU 83401^T^ | EF035074.2 | |
| *R. oryzicola* ZYY136^T^ | JX446583.2 | |
| *R. paknamense* L6-8^T^ | AB733647.1 | |
| R. *paranaense* PRF 35^T^ | EU488753.1 | |
| *R. phaseoli* ATCC 14482^T^ | EF141340.1 | |
| *R. pseudoryzae* J3-A127^T^ | DQ454123.3 | |
| *R. pusense* NRCPB10^T^ | FJ969841.2 | |
| *R. rosettiformans* W3^T^ | EU781656.2 | |
| *R. selenitireducens* B1^T^ | EF440185.1 | |
| *R.* *soli* DS-42^T^ | EF363715.1 | |
| *R. tibeticum* CCBAU 85039^T^ | EU256404.1 | |
| *R. tropici* CIAT 899^T^ | U89832.1 | |
| *R. vignae* CCBAU 05176^T^ | GU128881.1 | |
| ***Bradyrhizobium* sp. Moz 1** | **KY426346** | |
| ***B. elkanii* Moz 4** | **KY426347** | |
| ***Rhizobium* sp. Moz 8** | **KY426374** | |
| ***Bradyrhizobium* sp. Moz 9** | **KY426348** | |
| ***Rhizobium* sp. Moz 10** | **KY426375** | |
| ***Bradyrhizobium* sp. Moz 14** | **KY426349** | |
| ***Bradyrhizobium* sp. Moz 17** | **KY426350** | |
| ***B. elkanii* Moz 19** | **KY426351** | |
| ***Bradyrhizobium* sp. Moz 20** | **KY426352** | |
| ***B. elkanii* Moz 22** | **KY426353** | |
| ***B. japonicum* Moz 27** | **KY426354** | |
| ***Bradyrhizobium* sp. Moz 31** | **KY426355** | |
| ***Bradyrhizobium* sp. Moz 34** | **KY426356** | |
| ***Rhizobium* sp. Moz 36** | **KY426376** | |
| ***Rhizobium* sp. Moz 38** | **KY426377** | |
| ***Bradyrhizobium* sp. Moz 39** | **KY426357** | |
| ***Rhizobium* sp. Moz 42** | **KY426378** | |
| ***Bradyrhizobium* sp. Moz 44** | **KY426358** | |
| ***Bradyrhizobium* sp. Moz 45** | **KY426359** | |
| ***Bradyrhizobium* sp. Moz 48** | **KY426360** | |
| ***Rhizobium* sp. Moz 50** | **KY426379** | |
| ***Bradyrhizobium* sp. Moz 53** | **KY426361** | |
| ***Rhizobium* sp. Moz 55** | **KY426380** | |
| ***Bradyrhizobium* sp. Moz 56** | **KY426362** | |
| ***Rhizobium* sp. Moz 59** | **KY426381** | |
| ***Bradyrhizobium* sp. Moz 60** | **KY426363** | |
| ***B. japonicum* Moz 61** | **KY426364** | |
| ***Bradyrhizobium* sp. Moz 64** | **KY426365** | |
| ***Bradyrhizobium* sp. Moz 70** | **KY426366** | |
| ***Bradyrhizobium* sp. Moz 72** | **KY426367** | |
| ***Rhizobium* sp. Moz 73** | **KY426382** | |
| ***Rhizobium* sp. Moz 75** | **KY426383** | |
| ***Bradyrhizobium* sp. Moz 76** | **KY426368** | |
| ***Bradyrhizobium* sp. Moz 79** | **KY426369** | |
| ***Bradyrhizobium* sp. Moz 82** | **KY426370** | |
| ***Rhizobium* sp. Moz 88** | **KY426384** | |
| ***Rhizobium* sp. Moz 90** | **KY426385** | |
| ***Rhizobium* sp. Moz 92** | **KY426386** | |
| ***Rhizobium* sp. Moz 93** | **KY426387** | |
| ***Bradyrhizobium* sp. Moz 95** | **KY426371** | |
| ***Bradyrhizobium* sp. Moz 96** | **KY426372** | |
| ***Bradyrhizobium* sp. Moz 97** | **KY426373** | |
| ***Rhizobium* sp. Moz 99** | **KY426388** | |
| ***Rhizobium* sp. Moz 100** | **KY426389** | |

* Reference strains.

**Table S3**

Accession number of the sequences of the four housekeeping genes used in this study. The five isolates from Mozambique are indicated in bold.

| Strain | *dnaK* | *glnII* | *gyrB* | *recA* |
| --- | --- | --- | --- | --- |
| *B. arachidis* CCBAU 051107^T^ | JX437668.1 | HM107251.1 | JX437675.1 | HM107233.1 |
| *B. betae* LMG 21987^T^ | FM253303.1 | AB353733.1 | FM253217.1 | AB353734.1 |
| *B. canariense* LMG 22265^T^ | AY923047.1 | AY386765.1 | FM253220.1 | FM253177.1 |
| *B. cytisi* CTAW11^T^ | KF532219.1 | GU001594.1 | KF532653.1 | KF532947.1 |
| *B. daqingense* CCBAU 15774^T^ | LM994144.1 | HQ231301.1 | LM994190.1 | LM994320.1 |
| *B. diazoefficiens* SEMIA 5080 | FJ390997.1 | FJ391037.1 | JX867246.1 | FJ391157.1 |
| *B. diazoefficiens* USDA 110^T^ | NC_004463.1 | BA000040.2 | CP011360.1 | NC_004463.1 |
| *B. elkanii* SEMIA 5019 | FJ390990.1 | FJ391030.1 | NA | FJ391150.1 |
| *B. elkanii* SEMIA 587 | FJ390985.1 | FJ391025.1 | NA | FJ391145.1 |
| *B. elkanii* USDA 76^T^ | AY328392.1 | AY599117.1 | NZ KB900701.1 | KF532941.1 |
| *B. embrapense* SEMIA 6208^T^ | KP234519.2 | GQ160500.1 | HQ634891.1 | HQ634899.1 |
| *B. erythrophlei* CCBAU 53325^T^ | NA | KF114693.1 | KF114717.1 | KF114669.1 |
| *B. ferriligni* CCBAU 51502^T^ | NA | KJ818099.1 | KJ818102.1 | KJ818112.1 |
| *B. ganzhouense* RITF806^T^ | KP420023.1 | JX277110.1 | KP420022.1 | JX277144.1 |
| *B. guangxiense* CCBAU 53363^T^ | KC508974.1 | KC509033.1 | KC509082.1 | KC509279.1 |
| *B. huanghuaihaiense* CCBAU 23303^T^ | LM994145.1 | HQ231639.1 | LM994191.1 | HQ231595.1 |
| *B. ingae* BR 10250^T^ | KF927055.1 | KF927067.1 | KF927079.1 | KF927061.1 |
| *B. japonicum* SEMIA 5079 | FJ390996.1 | FJ391036.1 | NZ_CP007569.1 | FJ391156.1 |
| *B. japonicum* USDA 6^T^ | NC_017249.1 | AF169582.1 | AP012206.1 | AP012206.1 |
| *B. lablabi* CCBAU 23086^T^ | LM994147.1 | GU433498.1 | LM994192.1 | KF962710.1 |
| *B. liaoningense* LMG18230^T^ | AY923041.1 | AY386775.1 | FM253223.1 | AY591564.1 |
| *B. manausense* BR 3351^T^ | KF786001.1 | KF785986.1 | KF786000.1 | KF785992.1 |
| *B. neotropicale* BR 10247^T^ | KJ661693.1 | KJ661700.1 | KJ661707.1 | KJ661714.1 |
| *B. oligotrophicum* LMG 10732^T^ | KF962688.1 | JQ619233.1 | KF962697.1 | JQ619231.1 |
| *B. ottawaense* OO99^T^ | JF308816.1 | HQ587750.1 | HQ873179.1 | HQ587287.1 |
| *B. pachyrhizi* PAC48^T^ | LM994148.1 | FJ428201.1 | KF532651.1 | HM590777.1 |
| *B. paxllaeri* LMTR 21^T^ | AY923038.1 | KF896169.1 | KF896195.1 | JX943617.1 |
| *B. retamae* Ro19^T^ | KF896184.1 | KC247108.1 | KF896204.1 | KF962711.1 |
| *B. rifense* CTAW71^T^ | LM994143.1 | GU001604.1 | KC569466.1 | LM994317.1 |
| *B. subterraneum* 58 2-1^T^ | KP308157.1 | KM378484.1 | NA | KM378397.1 |
| *B. tropiciagri* SEMIA 6148^T^ | FJ391008.1 | FJ391048.1 | HQ634890.1 | FJ391168.1 |
| *B. valentinum* LmjM3^T^ | LLXX01000028.1 | JX518575.1 | LLXX01000044.1 | JX518589.2 |
| *B. vignae* 7-2^T^ | KR259951.1 | KM378443.1 | NA | KM378374.1 |
| *B. viridifuturi* SEMIA 690^T^ | KR149128.1 | KR149131.1 | KR149134.1 | KR149140.1 |
| [*B. yuanmingense* CCBAU 10071^T^](https://www.ncbi.nlm.nih.gov/nuccore/AY923039.1) | AY923039.1 | AY386780.1 | HE576508.1 | AY591566.1 |
| *Ensifer fredii* USDA 205^T^ | NZ_AUTC01000151.1 | AF169591.1 | AUTC01000138.1 | AJ294379.1 |
| ***B. elkanii* Moz 4** | **KY426390** | **KY426395** | **KY426400** | **KY426405** |
| ***B. elkanii* Moz 19** | **KY426391** | **KY426396** | **KY426401** | **KY426406** |
| ***B. elkanii* Moz 22** | **KY426392** | **KY426397** | **KY426402** | **KY426407** |
| ***B. japonicum* Moz 27** | **KY426393** | **KY426398** | **KY426403** | **KY426408** |
| ***B. japonicum* Moz 61** | **KY426394** | **KY426399** | **KY426404** | **KY426409** |

NA: not available.

**Table S4**

Nucleotide identity (%) comparisons among type strains *B. elkanii* USDA 76^T^, *B. japonicum* USDA 6T and *B. pachyrhizi* PAC48^T^ and five strains from Mozambique considering sequences of 16S rRNA, *dnaK*, *glnII*, *gyrB* and *recA* genes.

| Pair-wise comparison 16S rRNA | | | *dnaK* | *glnII* | *gyrB* | *recA* | Concatenated |
| --- | --- | --- | --- | --- | --- | --- | --- |
| *B. elkanii* USDA 76^T^ | *B. elkanii* Moz 4 | 99.7 | 99.5 | 99.1 | 95.7 | 100.0 | 98.4 |
| *B. elkanii* USDA 76 ^T^ | *B. elkanii* Moz 19 | 100.0 | 100.0 | 99.3 | 100.0 | 99.7 | 99.7 |
| *B. elkanii* USDA 76 ^T^ | *B. elkanii* Moz 22 | 100.0 | 100.0 | 99.1 | 100.0 | 100.0 | 99.7 |
| *B. elkanii* USDA 76 ^T^ | *B. japonicum* Moz 27 | 95.2 | 88.3 | 88.5 | 91.6 | 92.5 | 90.3 |
| *B. elkanii* USDA 76 ^T^ | *B. japonicum* Moz 61 | 96.3 | 88.3 | 88.5 | 92.1 | 92.2 | 90.4 |
| *B. elkanii* Moz 4 | *B. elkanii* Moz 19 | 99.7 | 99.5 | 99.7 | 95.7 | 99.7 | 98.5 |
| *B. elkanii* Moz 4 | *B. elkanii* Moz 22 | 99.7 | 99.5 | 99.3 | 95.7 | 100.0 | 98.5 |
| *B. elkanii* Moz 4 | *B. japonicum* Moz 27 | 95.0 | 88.7 | 88.5 | 91.4 | 92.5 | 90.3 |
| *B. elkanii* Moz 4 | *B. japonicum* Moz 61 | 96.0 | 88.7 | 88.5 | 91.8 | 92.2 | 90.4 |
| *B. elkanii* Moz 19 | *B. elkanii* Moz 22 | 100.0 | 100.0 | 99.3 | 100.0 | 99.7 | 99.7 |
| *B. elkanii* Moz 19 | *B. japonicum* Moz 27 | 95.2 | 88.3 | 88.7 | 91.6 | 92.8 | 90.5 |
| *B. elkanii* Moz 19 | *B. japonicum* Moz 61 | 96.3 | 88.3 | 88.7 | 92.1 | 92.5 | 90.5 |
| *B. elkanii* Moz 22 | *B. japonicum* Moz 27 | 95.2 | 88.3 | 88.7 | 91.6 | 92.5 | 90.4 |
| *B. elkanii* Moz 22 | *B. japonicum* Moz 61 | 96.3 | 88.3 | 88.7 | 92.1 | 92.2 | 90.5 |
| *B. japonicum* Moz 27 | *B. japonicum* Moz 61 | 98.6 | 100.0 | 100.0 | 99.2 | 99.2 | 99.5 |
| *B. japonicum* USDA 6^T^ | *B. elkanii* Moz 4 | 96.0 | 88.7 | 88.5 | 91.8 | 92.2 | 90.4 |
| *B. japonicum* USDA 6^T^ | *B. elkanii* Moz 19 | 96.3 | 88.3 | 88.7 | 92.1 | 92.5 | 90.5 |
| *B. japonicum* USDA 6^T^ | *B. elkanii* Moz 22 | 96.3 | 88.3 | 88.7 | 92.1 | 92.2 | 90.5 |
| *B. japonicum* USDA 6^T^ | *B. japonicum* Moz 27 | 98.6 | 100.0 | 100.0 | 99.2 | 99.2 | 99.5 |
| *B. japonicum* USDA 6^T^ | *B. japonicum* Moz 61 | 100.0 | 100.0 | 100.0 | 100.0 | 100.0 | 100.0 |
| *B. japonicum* USDA 6^T^ | *B. elkanii* USDA 76^T^ | 96.3 | 88.3 | 88.5 | 92.1 | 92.2 | 90.4 |
| *B. pachyrhizi* PAC48^T^ | *B. elkanii* Moz 4 | 99.7 | 99.5 | 95.8 | 95.2 | 95.4 | 96.1 |
| *B. pachyrhizi* PAC48^T^ | *B. elkanii* Moz 19 | 100.0 | 100.0 | 96.0 | 98.3 | 95.2 | 97.0 |
| *B. pachyrhizi* PAC48^T^ | *B. elkanii* Moz 22 | 100.0 | 100.0 | 95.8 | 98.3 | 95.4 | 97.0 |
| *B. pachyrhizi* PAC48^T^ | *B. japonicum* Moz 27 | 95.2 | 88.3 | 87.9 | 91.8 | 91.7 | 90.0 |
| *B. pachyrhizi* PAC48^T^ | *B. japonicum* Moz 61 | 96.3 | 88.3 | 87.9 | 92.3 | 91.4 | 90.1 |
| *B. pachyrhizi* PAC48^T^ | *B. elkanii* USDA 76^T^ | 100.0 | 100.0 | 95.8 | 98.3 | 95.4 | 97.0 |
| *B. pachyrhizi* PAC48^T^ | *B. japonicum* USDA 6^T^ | 96.3 | 88.3 | 87.9 | 92.3 | 91.4 | 90.1 |

NA – not available.

##### Table S5

##### Nodule number (NN, n° plant^−1^) and dry weight (NDW, mg plant^−1^), shoot dry weight (SDW, g plant^−1^), total N accumulation in shoots (TNS, mg plant^−1^) and relative effectiveness (RE, %) of soybean, cultivar BRS 133, inoculated with 87 isolates from Mozambique and five reference strains, *B. elkanii* SEMIA 587 and SEMIA 5019, *B. japonicum* SEMIA 5079, and *B. diazoefficiens* SEMIA 5080 and USDA 110 screened in a greenhouse trial in Londrina, Brazil, in 2014.

| Isolate | Source | Species name ^1^ | NN^2^ | NDW | SDW | TNS | RE^3^ |
| --- | --- | --- | --- | --- | --- | --- | --- |
| Moz 1 | Ntengo | *Bradyrhizobium* sp. | 79.9 | 453.80 | 4.0 | 98.93 | 96.3 |
| Moz 2 ^¥^ | Ntengo | *Bradyrhizobium* sp. | 56.3 | 380.43 | 3.4 | 73.17 | 80.4 |
| Moz 3 | Ntengo | *Bradyrhizobium* sp. | 81.4 | 465.54 | 4.9 | 105.32 | 115.2 |
| Moz 4^* ¥¥^ | Ntengo | *Bradyrhizobium* sp. | 63.4 | 508.00 | 5.7 | 142.03 | 135.5 |
| Moz 5 | Ntengo | *Bradyrhizobium* sp. | 73.3 | 557.48 | 5.4 | 131.29 | 126.3 |
| Moz 6^*^ | Ntengo | *Bradyrhizobium* sp. | 103.6 | 498.81 | 4.9 | 104.15 | 116.9 |
| Moz 7 | Ntengo | *Bradyrhizobium* sp. | 60.6 | 392.05 | 3.9 | 76.36 | 95.7 |
| Moz 8 | Ntengo | *Agrobacterium* sp. | 38.3 | 241.22 | 2.7 | 40.80 | 64.0 |
| Moz 9 | Ntengo | *Bradyrhizobium* sp. | 36.4 | 191.06 | 2.2 | 36.93 | 51.1 |
| Moz 10 | Ntengo | *Rhizobium* sp. | 7.0 | 29.14 | 0.8 | 6.51 | 18.2 |
| Moz 11 | Ntengo | *Bradyrhizobium* sp. | 6.0 | 20.19 | 0.8 | 4.98 | 18.6 |
| Moz 14 | Ntengo | *Bradyrhizobium* sp. | 10.0 | 27.53 | 1.0 | 9.20 | 24.3 |
| Moz 15 | Ntengo | *Bradyrhizobium* sp. | 86.5 | 531.65 | 5.0 | 108.19 | 107.7 |
| Moz 17^*^ | Ntengo | *Bradyrhizobium* sp. | 88.0 | 586.10 | 5.8 | 123.32 | 139.4 |
| Moz 18 | Ntengo | *Bradyrhizobium* sp. | 102.6 | 527.30 | 5.0 | 114.44 | 120.6 |
| Moz 19^*^ | Ntengo | *Bradyrhizobium* sp. | 106.8 | 539.89 | 5.1 | 139.19 | 124.0 |
| Moz 20 | Ntengo | *Bradyrhizobium* sp. | 78.1 | 594.66 | 5.3 | 131.68 | 128.1 |
| Moz 22^*^ | Nkhame | *Bradyrhizobium* sp. | 76.0 | 474.31 | 6.2 | 152.70 | 135.3 |
| Moz 23 | Nkhame | *Bradyrhizobium* sp. | 79.9 | 595.50 | 5.8 | 146.57 | 137.4 |
| Moz 24^*^ | Nkhame | *Bradyrhizobium* sp. | 102.0 | 588.26 | 5.8 | 146.78 | 138.4 |
| Moz 25 | Nkhame | *Bradyrhizobium* sp. | 65.8 | 478.60 | 5.3 | 141.01 | 114.1 |
| Moz 26 | Nkhame | *Bradyrhizobium* sp. | 70.8 | 520.29 | 5.3 | 143.88 | 126.7 |
| Moz 27^*^ | Nkhame | *Bradyrhizobium* sp. | 65.5 | 493.08 | 5.2 | 146.15 | 129.5 |
| Moz 28 | Nkhame | *Bradyrhizobium* sp. | 77.9 | 601.06 | 5.5 | 136.19 | 129.1 |
| Moz 29 | Nkhame | Bradyrhizobium sp. | 77.5 | 465.88 | 3.1 | 61.75 | 73.2 |
| Moz 30 | Nkhame | *Bradyrhizobium* sp. | 46.1 | 340.00 | 3.5 | 58.64 | 84.6 |
| Moz 31 | Nkhame | *Bradyrhizobium* sp. | 82.3 | 447.43 | 3.1 | 57.18 | 72.9 |
| Moz 32 | Nkhame | *Bradyrhizobium* sp. | 84.8 | 511.89 | 4.6 | 121.66 | 109.8 |
| Moz 33 | Nkhame | *Bradyrhizobium* sp. | 82.6 | 527.56 | 4.7 | 113.38 | 112.3 |
| Moz 31 | Nkhame | *Bradyrhizobium* sp. | 82.3 | 447.43 | 3.1 | 57.18 | 72.9 |
| Moz 35 | Nkhame | *Bradyrhizobium* sp. | 68.1 | 538.13 | 5.4 | 144.08 | 129.7 |
| Moz 36 | Nkhame | *Rhizobium* sp. | 9.4 | 29.68 | 0.8 | 7.19 | 19.1 |
| Moz 37 | Nkhame | *Bradyrhizobium* sp. | 87.3 | 592.80 | 4.5 | 90.67 | 108.4 |
| Moz 38^*^ | Nkhame | *Rhizobium* sp. | 73.0 | 541.83 | 4.0 | 100.71 | 96.4 |
| Moz 39^*^ | Nkhame | *Bradyrhizobium* sp. | 83.5 | 638.99 | 5.0 | 152.92 | 119.8 |
| Moz 40^*^ | Nkhame | *Bradyrhizobium* sp. | 60.6 | 512.78 | 5.0 | 142.85 | 117.2 |
| Moz 41 | Nkhame | *Bradyrhizobium* sp. | 70.8 | 475.45 | 4.3 | 110.42 | 92.3 |
| Moz 42 | Nkhame | *Rhizobium* sp. | 64.3 | 488.68 | 3.8 | 107.68 | 88.9 |
| Moz 43 | Ruace | *Bradyrhizobium* sp. | 30.0 | 64.43 | 1.0 | 10.08 | 23.0 |
| Moz 44 | Ruace | *Bradyrhizobium* sp. | 9.8 | 19.71 | 0.7 | 4.74 | 17.2 |
| Moz 45 | Ruace | *Bradyrhizobium* sp. | 8.9 | 32.30 | 0.8 | 8.27 | 18.5 |
| Moz 46 | Ruace | *Bradyrhizobium* sp. | 9.4 | 30.85 | 0.6 | 3.93 | 15.3 |
| Moz 48 | Ruace | *Bradyrhizobium* sp. | 19.3 | 82.31 | 1.1 | 15.67 | 26.7 |
| Moz 50 | Ruace | *Rhizobium* sp. | 5.5 | 18.93 | 0.8 | 5.76 | 20.5 |
| Moz 52 | Ruace | *Bradyrhizobium* sp. | 18.8 | 76.99 | 1.1 | 14.04 | 24.7 |
| Moz 53 | Ruace | *Bradyrhizobium* sp. | 25.6 | 81.10 | 0.9 | 11.16 | 21.1 |
| Moz 55 | Ruace | *Rhizobium* sp. | 11.4 | 55.44 | 0.9 | 10.98 | 21.2 |
| Moz 56 | Ruace | *Bradyrhizobium* sp. | 11.8 | 54.59 | 0.8 | 6.97 | 18.3 |
| Moz 57 | Mutequelesse | *Bradyrhizobium* sp. | 58.4 | 435.56 | 4.4 | 127.64 | 105.3 |
| Moz 58 | Mutequelesse | *Bradyrhizobium* sp. | 59.6 | 452.94 | 5.0 | 137.59 | 106.6 |
| Moz 59 | Mutequelesse | *Rhizobium* sp. | 37.4 | 310.11 | 2.9 | 53.69 | 70.4 |
| Moz 60 | Mutequelesse | *Bradyrhizobium* sp. | 90.9 | 399.29 | 4.5 | 114.61 | 107.3 |
| Moz 61^*^ | Mutequelesse | *Bradyrhizobium* sp. | 63.8 | 440.28 | 4.9 | 146.57 | 116.8 |
| Moz 62^*^ | Mutequelesse | *Bradyrhizobium* sp. | 98.5 | 593.78 | 5.4 | 147.83 | 129.6 |
| Moz 63 | Mutequelesse | *Bradyrhizobium* sp. | 86.3 | 538.11 | 5.0 | 134.41 | 120.1 |
| Moz 64 | Muriaze | *Bradyrhizobium* sp. | 81.9 | 276.54 | 2.6 | 68.17 | 60.0 |
| Moz 65 | Muriaze | *Bradyrhizobium* sp. | 29.0 | 181.61 | 1.8 | 22.25 | 36.8 |
| Moz 66 | Muriaze | *Bradyrhizobium* sp. | 21.5 | 51.89 | 0.9 | 18.30 | 19.9 |
| Moz 67 | Muriaze | *Bradyrhizobium* sp. | 17.8 | 43.08 | 1.0 | 10.04 | 23.1 |
| Moz 69 | Muriaze | *Rhizobium* sp. | 10.8 | 40.45 | 1.1 | 18.24 | 24.1 |
| Moz 70 | Muriaze | *Bradyrhizobium* sp. | 38.0 | 161.78 | 1.5 | 32.01 | 34.9 |
| Moz 71 | Muriaze | *Bradyrhizobium* sp. | 21.6 | 82.16 | 1.5 | 16.05 | 35.1 |
| Moz 72 | Muriaze | *Bradyrhizobium* sp. | 26.5 | 89.63 | 1.4 | 19.03 | 33.5 |
| Moz 73 | Muriaze | *Rhizobium* sp. | 23.6 | 63.90 | 0.9 | 12.94 | 21.6 |
| Moz 74 | Muriaze | *Bradyrhizobium* sp. | 15.5 | 77.51 | 1.2 | 18.56 | 29.4 |
| Moz 75 | Muriaze | *Rhizobium* sp. | 16.1 | 53.29 | 0.8 | 13.35 | 19.3 |
| Moz 76 | Muriaze | *Bradyrhizobium* sp. | 23.0 | 75.06 | 1.3 | 17.78 | 31.3 |
| Moz 77 | Muriaze | *Bradyrhizobium* sp. | 27.0 | 165.80 | 2.3 | 37.51 | 53.3 |
| Moz 78 | Sussundenga | *Bradyrhizobium* sp. | 5.0 | 36.74 | 0.7 | 7.08 | 15.6 |
| Moz 79 | Sussundenga | *Bradyrhizobium* sp. | 6.8 | 47.30 | 0.8 | 7.53 | 18.6 |
| Moz 80 | Sussundenga | *Bradyrhizobium* sp. | 7.6 | 42.03 | 0.9 | 10.36 | 21.5 |
| Moz 81 | Sussundenga | *Bradyrhizobium* sp. | 10.9 | 35.96 | 0.8 | 8.24 | 18.5 |
| Moz 82 | Sussundenga | *Bradyrhizobium* sp. | 11.0 | 38.88 | 0.8 | 8.03 | 20.3 |
| Moz 85 | Sussundenga | *Rhizobium* sp. | 6.6 | 22.24 | 0.9 | 7.22 | 21.6 |
| Moz 86 | Sussundenga | *Rhizobium* sp. | 15.0 | 79.25 | 1.0 | 19.25 | 23.2 |
| Moz 87 | Sussundenga | *Rhizobium* sp. | 63.6 | 309.55 | 3.2 | 97.65 | 76.3 |
| Moz 88 | Sussundenga | *Rhizobium* sp. | 17.1 | 31.28 | 0.9 | 9.13 | 22.2 |
| Moz 90 | Sussundenga | *Rhizobium* sp. | 15.4 | 88.21 | 1.1 | 12.29 | 25.9 |
| Moz 91 | Sussundenga | *Rhizobium* sp. | 20.9 | 92.43 | 1.3 | 18.14 | 28.5 |
| Moz 92 | Zembe | *Rhizobium* sp. | 42.5 | 191.81 | 1.8 | 42.97 | 43.6 |
| Moz 93 | Zembe | *Rhizobium* sp. | 12.5 | 39.39 | 0.8 | 8.12 | 18.3 |
| Moz 94 | Zembe | *Rhizobium* sp. | 10.4 | 26.95 | 0.8 | 8.27 | 18.3 |
| Moz 95^*^ | Zembe | *Bradyrhizobium* sp. | 68.3 | 397.79 | 4.2 | 102.18 | 98.9 |
| Moz 96 | Zembe | *Bradyrhizobium* sp. | 10.3 | 41.55 | 0.9 | 13.87 | 22.2 |
| Moz 97 | Zembe | *Bradyrhizobium* sp. | 68.8 | 352.67 | 3.6 | 92.65 | 88.0 |
| Moz 99 | Zembe | *Rhizobium* sp. | 31.4 | 70.24 | 1.0 | 13.14 | 23.3 |
| Moz 100 | Zembe | *Rhizobium* sp. | 10.3 | 41.03 | 1.2 | 16.53 | 29.3 |
| Reference strains | | | | | | | |
| USDA 110 | EUA | *B. diazoefficiens* | 61.5 | 408.81 | 5.4 | 140.20 | 127.9 |
| SEMIA 587 | Brasil | *Bradyrhizobium elkanii* | 59.8 | 265.06 | 3.9 | 84.60 | 93.9 |
| SEMIA 5019 | Brasil | *Bradyrhizobium elkanii* | 56.4 | 513.58 | 5.0 | 119.28 | 118.7 |
| SEMIA 5079 | Brasil | *B. japonicum* | 73.4 | 350.91 | 3.4 | 89.16 | 81.5 |
| SEMIA 5080 | Brasil | *B. diazoefficiens* | 81.6 | 391.29 | 3.7 | 77.31 | 86.4 |
| Control + N |  |  | 0.0 | 0.00 | 4.3 | 98.34 | 100.0 |
| Control - N |  |  | 0.0 | 0.00 | 0.7 | 4.75 | 167 |
| C.V. (%) |  |  | 31.1 | 22.40 | 20.0 | 13.14 | 10.4 |

^1^ Based on the analysis of 16S rRNA (Figs. 2 and 3).

^2^ Means of four replicates.

^3^Expressed as the percentage of shoot dry weight of plants supplied N (Control + N) compared to treatment with inoculant.^¥^ Isolates highlighted in yellow had inferior symbiotic effectiveness than all the reference strains.

^¥^ Isolates highlighted in green had better symbiotic effectiveness than reference strain USDA 110.

^*^ Isolates selected for the second greenhouse trial.

##### Table S6

##### Relative effectiveness (range, mean and standard deviation) of soybean, cultivar BRS 133, inoculated with 87 rhizobial isolates from Mozambique. Strains from BOX – PCR clusters and five commercial, *B. elkanii* SEMIA 587 and SEMIA 5019, *B. japonicum* SEMIA 5079, *B. diazoefficiens* SEMIA 5080 and USDA 110 inoculated on soybean, cultivar BRS 133, and screened for N_2_-fixation in a greenhouse trial in Londrina, Brazil, in 2014.

| Clusters^1^  Number of isolates | | | RE^2^ Range (%) | RE Mean (%) | RE Std deviation (%) |
| --- | --- | --- | --- | --- | --- |
| 1 | 2 | | 72.9 – 73.2 | 73.0 | 0.2 |
| 2 | 1 | |  | 129.5 |  |
| 3 | 1 | |  | 88.0 |  |
| 4 | 1 | |  | 98.9 |  |
| 5 | 1 | |  | 18.2 |  |
| 6 | 1 | |  | 96.4 |  |
| 7 | 3 | | 21.6 – 76.3 | 40.0 | 31.4 |
| 8 | 2 | | 21.1 – 24.7 | 22.9 | 2.6 |
| 9 | 1 | |  | 18.3 |  |
| 10 | 1 | |  | 48.8 |  |
| 11 | 2 | | 18.5 – 20.3 | 19.4 | 1.3 |
| 12 | 2 | | 15.3 – 26.7 | 21.0 | 8.0 |
| 13 | 2 | | 107.3 – 116.8 | 112.0 | 6.7 |
| 14 | 4 | | 92.3 –119.8 | 109.4 | 12.4 |
| 15 | 15 | | 84.6 – 138.4 | 120.5 | 14.9 |
| 16 | 4 | | 107.7 – 139.4 | 122.9 | 13.1 |
| 17 | 5 | | 80.4 – 116.9 | 100.9 | 15.2 |
| 18 | 2 | | 126.3 – 135.5 | 130.9 | 6.5 |
| 19 | 4 | | 19.9 – 60.0 | 35.0 | 18.2 |
| 20 | 1 | |  | 70.4 |  |
| 21 | 1 | |  | 34.9 |  |
| 22 | 3 | | 23.2 – 28.5 | 25.9 | 2.6 |
| 23 | 1 | |  | 21.2 |  |
| 24 | 1 | |  | 21.6 |  |
| 25 | 3 | | 15.6 – 21.5 | 18.6 | 2.9 |
| 26 | 1 | |  | 20.5 |  |
| 27 | 2 | | 31.3 – 53.3 | 42.3 | 15.6 |
| 28 | 1 | |  | 19.3 |  |
| 29 | 1 | |  | 88.9 |  |
| 30 | 2 | | 24.1 – 43.6 | 33.8 | 13.8 |
| 31 | 1 | |  | 18.3 |  |
| 32 | 2 | | 18.3 – 29.3 | 23.8 | 7.8 |
| 33 | 1 | |  | 64.0 |  |
| 34 | 2 | | 18.6 – 51.1 | 34.9 | 23.0 |
| 35 | 1 | |  | 24.3 |  |
| 36 | 1 | |  | 17.2 |  |
| 37 | 3 | | 29.4 – 35.1 | 32.7 | 3.0 |
| 38 | 2 | | 18.5 – 23.0 | 20.8 | 3.2 |
| 39 | 1 | |  | 19.1 |  |
| 40 | 1 | |  | 22.2 |  |
| 41 | 1 | |  | 23.3 |  |
| Reference strains | | | |  |  |
| *B. diazoefficiens* USDA 110 | |  | | 127.9 |  |
| *B. elkanii* SEMIA 587 | |  | | 93.9 |  |
| *B. elkanii* SEMIA 5019 | |  | | 118.8 |  |
| *B. japonicum* SEMIA 5079 | |  | | 81.5 |  |
| *B. diazoefficiens* SEMIA 5080 |  |  | | 86.4 |  |

^1^ Phylogenetic cluster as defined by BOX-PCR analysis (Fig. 1).

^2^ Each isolate represented by four replications and RE expressed as the percentage of shoot dry weight of plants supplied with N (Control + N).


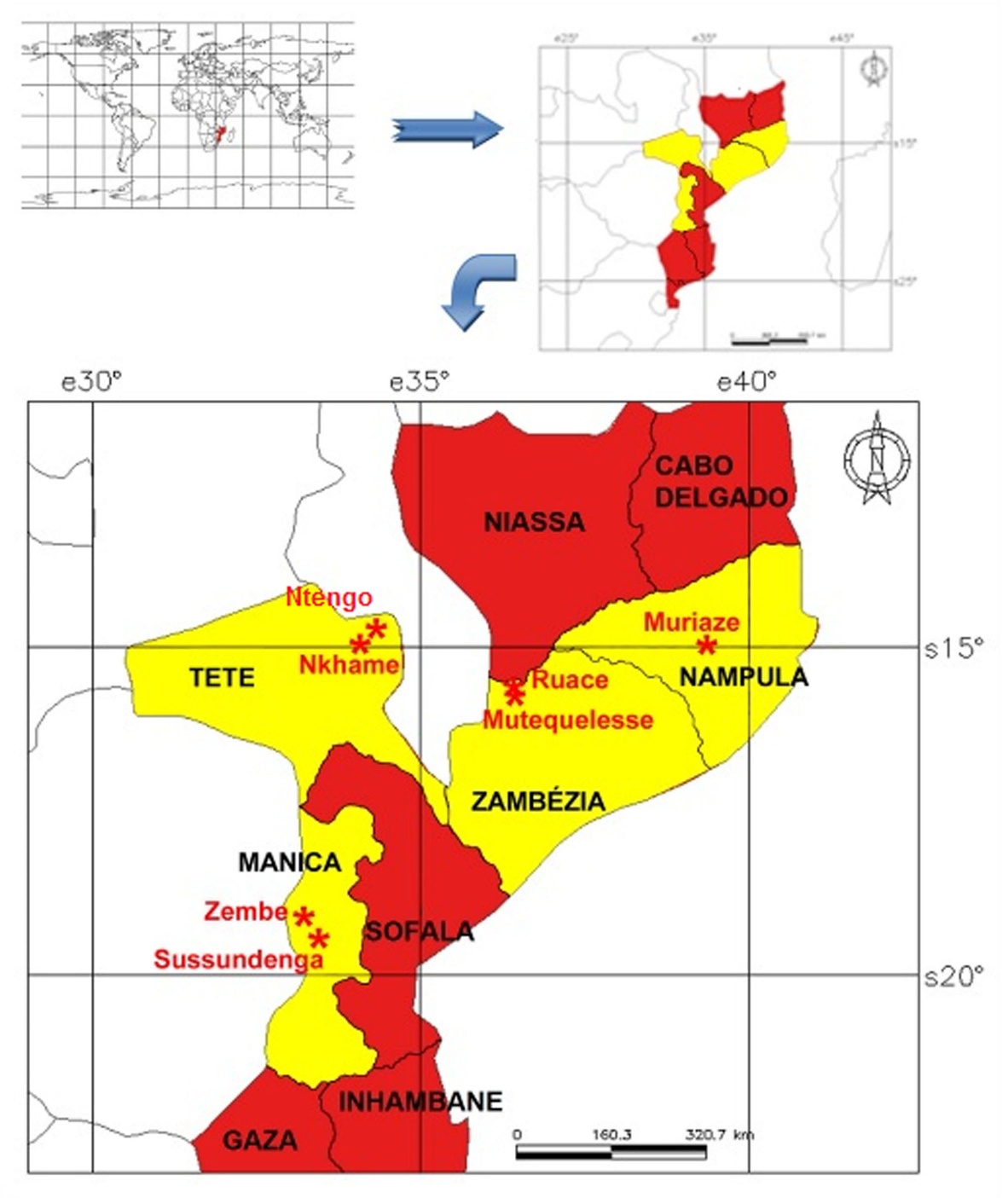


**Fig. S1**

Locations of the sites from where promiscuous soybean nodules were sampled in Manica, Nampula, Tete and Zambézia provinces, which represent the major soybean production area in Mozambique. Map with courtesy from Dr. Osvaldo Coelho Pereira Neto (Universidade Estadual de Londrina).


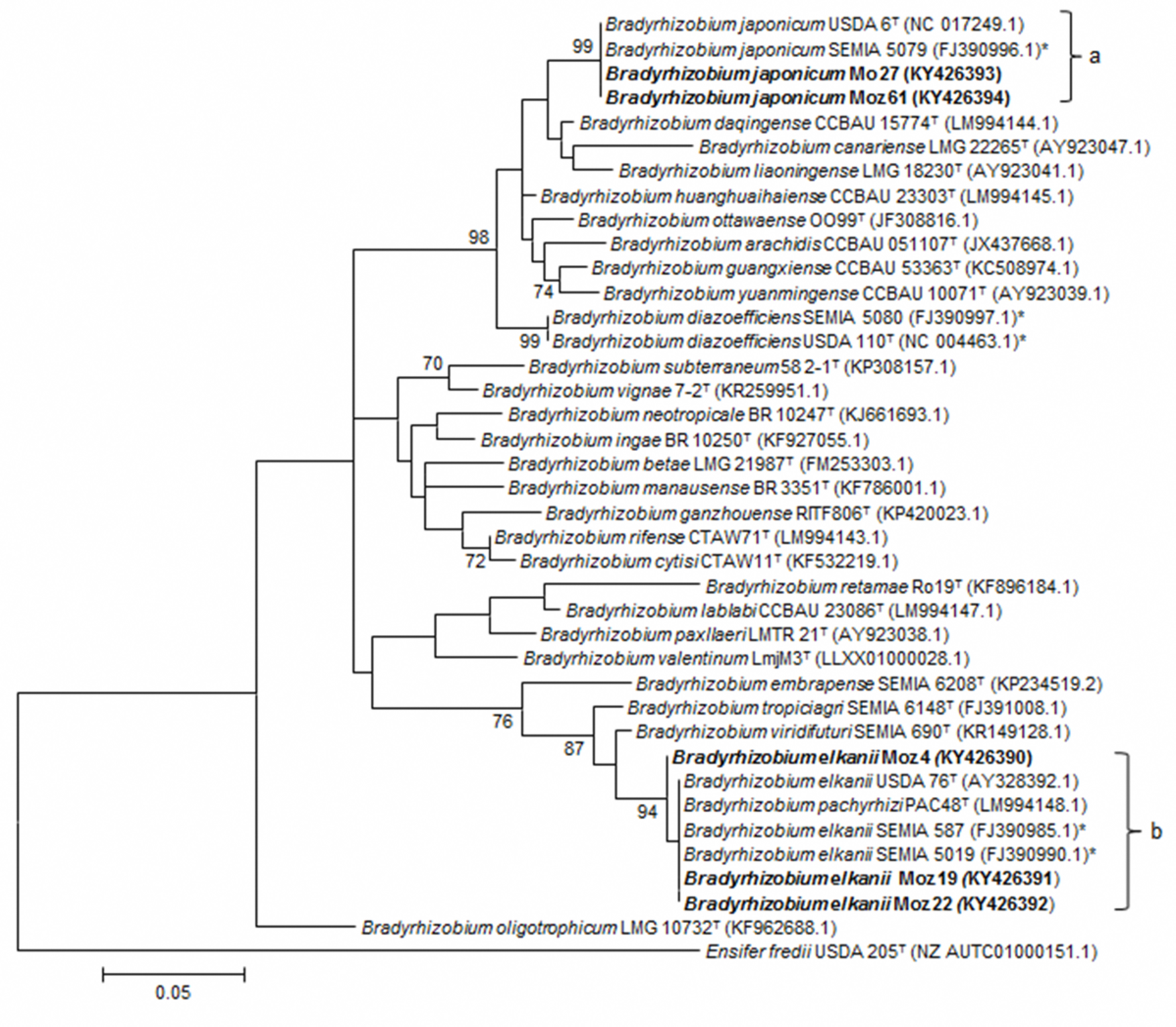


**Fig. S2**

Maximum - likelihood phylogeny based on *dnaK* gene sequences (223 bp) showing the relationships among five rhizobial isolates from Mozambique (in bold) with type (^T^) and reference strains used in commercial inoculants, *B. elkanii* SEMIA 587 and SEMIA 5019, *B. japonicum* SEMIA 5079, and *B. diazoefficiens* SEMIA 5080 and USDA 110 (with an asterix). *Ensifer* *fredii* USDA 205^T^ was inluded as an outgroup. Only bootstrap confidence levels > 70% are shown at the internodes. The scale bar indicates 5 substitutions per 100 nucleotides; a and b represent the clustering of the five best nitrogen fixer strains from Mozambique.


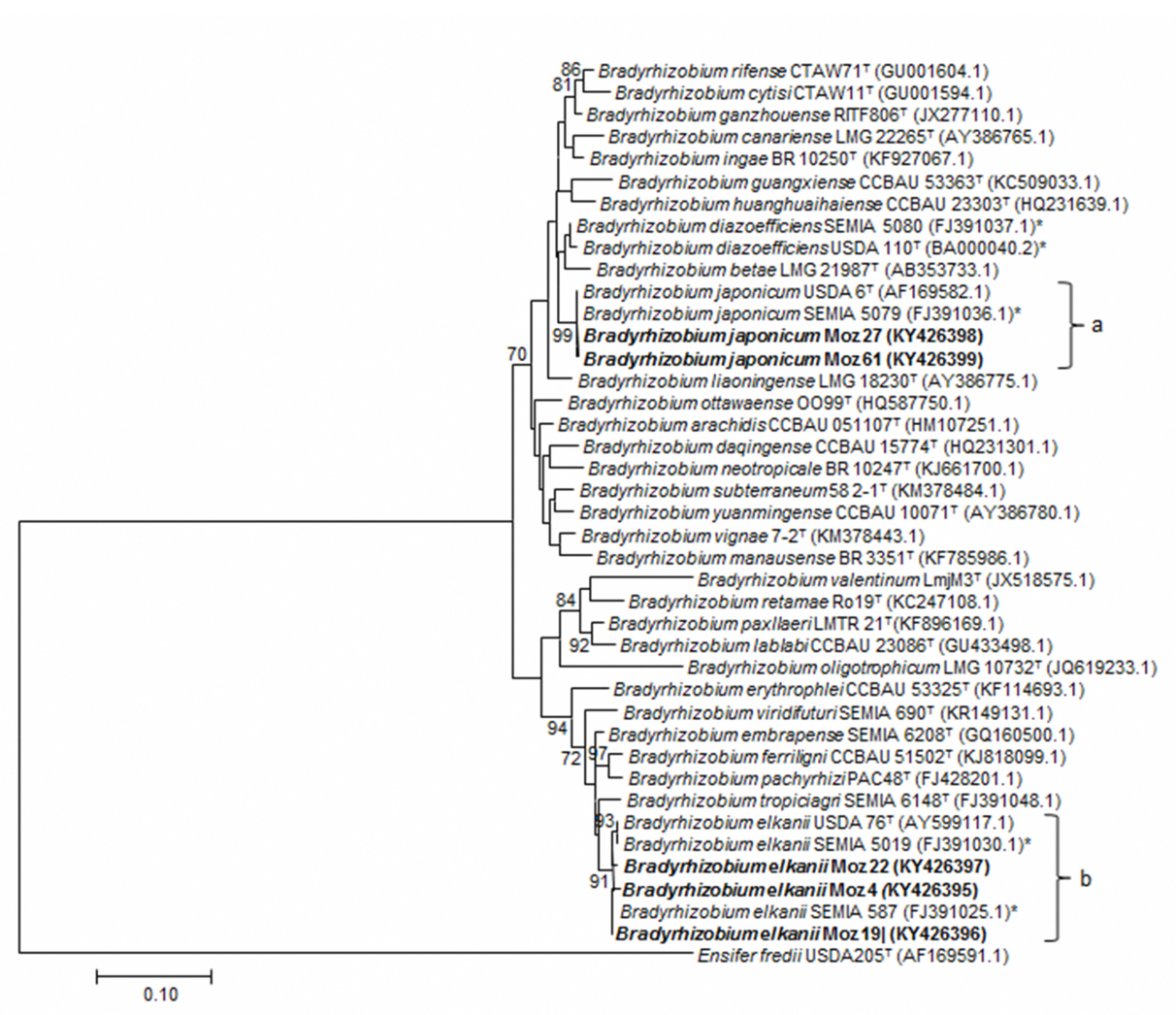


**Fig. S3**

Maximum - likelihood phylogeny based on *glnII* gene sequences (480 bp) showing the relationships among five rhizobial isolates from Mozambique (in bold) with type (^T^) and reference strains used in commercial inoculants, *B. elkanii* SEMIA 587 and SEMIA 5019, *B. japonicum* SEMIA 5079, and *B. diazoefficiens* SEMIA 5080 and USDA 110 (with an asterix). *Ensifer* *fredii* USDA 205^T^ was inluded as an outgroup. Only bootstrap confidence levels > 70% are shown at the internodes. The scale bar indicates 1 substitution per 10 nucleotides; a and b represent the clustering of the five best nitrogen fixer strains from Mozambique.


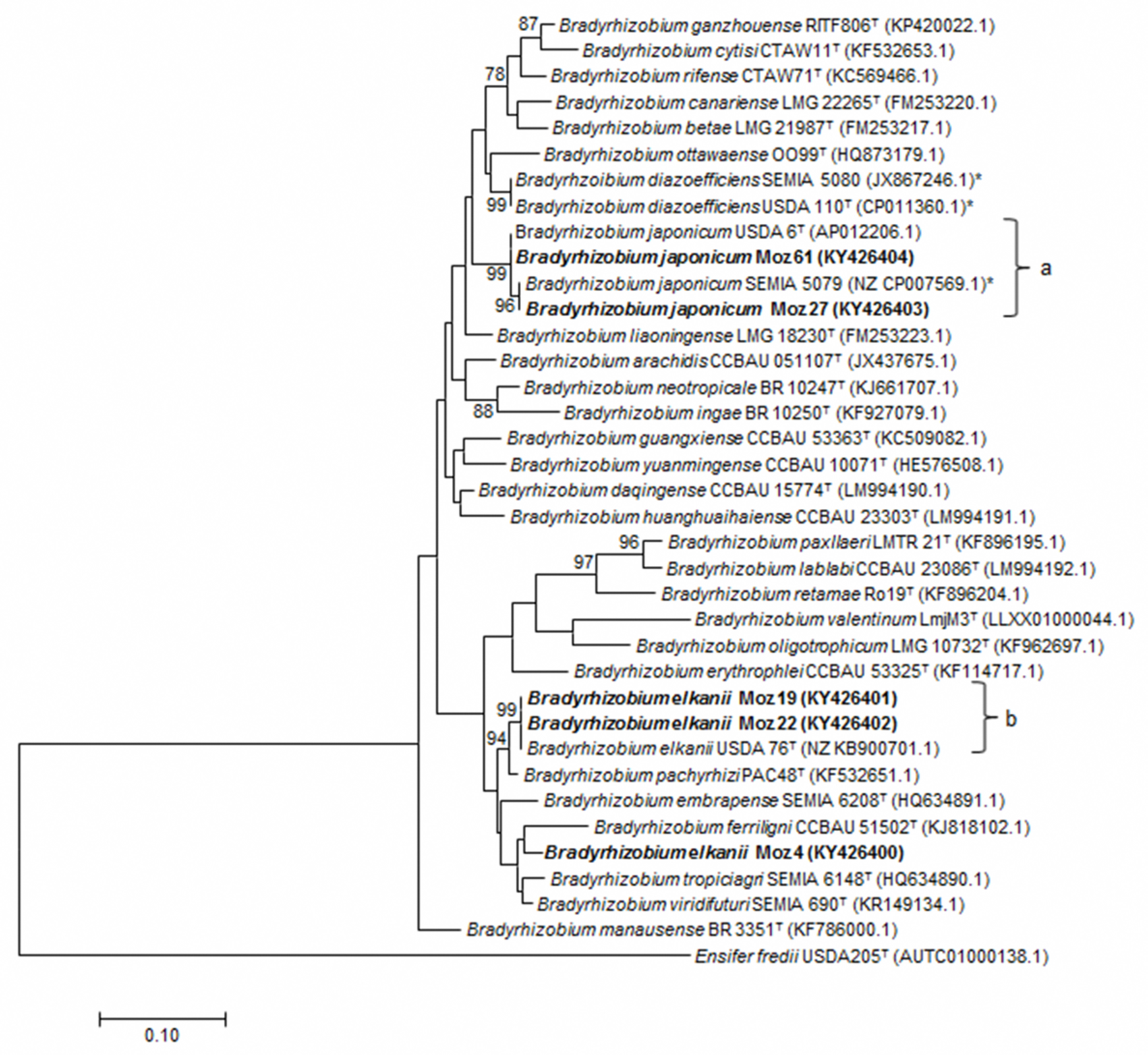


**Fig. S4**

Maximum - likelihood phylogeny based on *gyrB* gene sequences (419 bp) showing the relationships among five rhizobial isolates from Mozambique (in bold) with type (^T^) and reference strains used in commercial inoculants, *B. elkanii* SEMIA 587 and SEMIA 5019, *B. japonicum* SEMIA 5079, and *B. diazoefficiens* SEMIA 5080 and USDA 110 (with an asterix). *Ensifer* *fredii* USDA 205^T^ was inluded as an outgroup. Only bootstrap confidence levels > 70% are shown at the internodes. The scale bar indicates 1 substitution per 10 nucleotides; a and b represent the clustering of the five best nitrogen fixer strains from Mozambique.


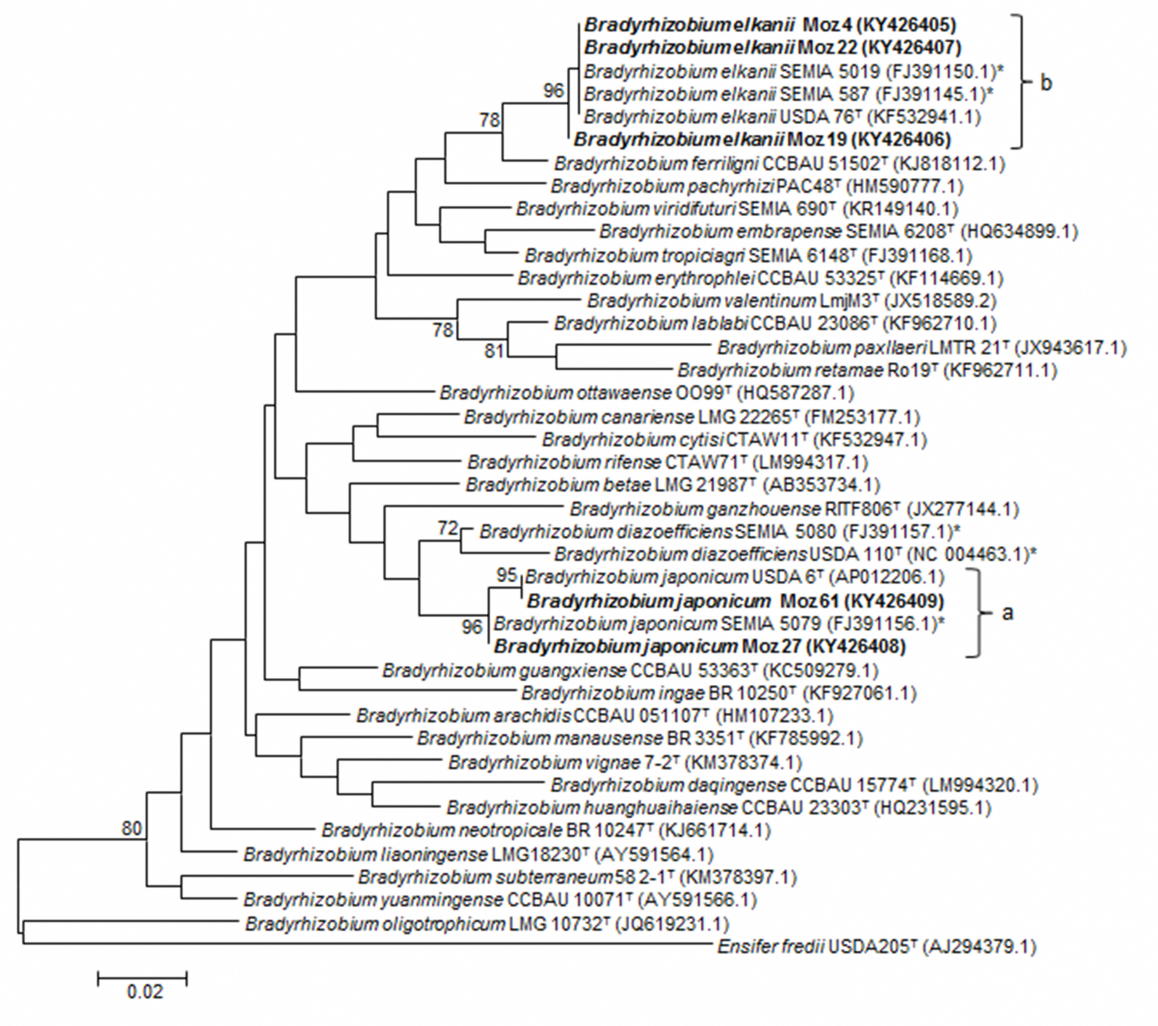


**Fig. S5**

Maximum - likelihood phylogeny based on *recA* gene sequences (375 bp) showing the relationships among five rhizobial isolates from Mozambique (in bold) with type (^T^) and reference strains used in commercial inoculants, *B. elkanii* SEMIA 587 and SEMIA 5019, *B. japonicum* SEMIA 5079, and *B. diazoefficiens* SEMIA 5080 and USDA 110 (with an asterix). *Ensifer* *fredii* USDA 205^T^ was inluded as an outgroup. Only bootstrap confidence levels > 70% are shown at the internodes. The scale bar indicates 2 substitution per 100 nucleotides; a and b represent the clustering of the five best nitrogen fixer strains from Mozambique.

**
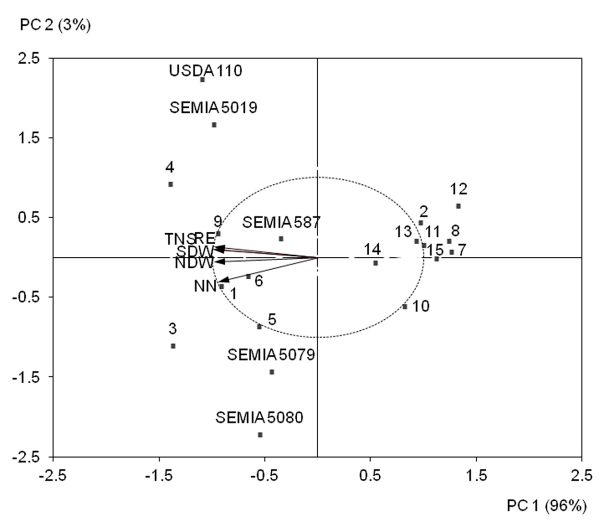
****Fig. S6**

Principal component analysis exploring the relationships among sampling sites as sources of high or poor performing representative isolates. Reference strains *B. elkanii* SEMIA 587 and SEMIA 5019, *B. japonicum* SEMIA 5079, and *B. diazoefficiens* SEMIA 5080 and USDA 110 represent the ideal sources of good performing strains. Sources of isolates and strains are compared considering variables NN and NDW, SDW, TNS and RE. Number represent sampling sites: 1 – Ntengo­_1_; 2 – Ntengo_2_; 3 – Ntengo_3_; 4 – Khame_1_; 5 – Khame_2_; 6 – Khame_3_; 7 – Ruace_1_; 8 – Ruace_2;_ 9 – Mutequelesse; 10 – Muriaze_1_; 11 – Muriaze_2_; 12 – Sussundenga_1_; 13 – Sussundenga_2_; 14 – Zembe_1_; 15 – Zembe_2_.
